# The dinosaurs that weren’t: osteohistology supports giant ichthyosaur affinity of enigmatic large bone segments from the European Rhaetian

**DOI:** 10.7717/peerj.17060

**Published:** 2024-04-09

**Authors:** Marcello Perillo, P Martin Sander

**Affiliations:** 1 Section Paleontology, Institute of Geosciences, Rheinische Friedrich-Wilhelms Universität Bonn, Bonn, Germany; 2 The Dinosaur Institute, Natural History Museum of Los Angeles County, Los Angeles, CA, United States of America

**Keywords:** European fossil deposits, Late Triassic, Rhaetian, Giant ichthyosaurs, Shastasauridae, Osteohistology, Cranial osteohistology, Metaplastic ossification, Bone specialization, Archosaur osteohistology

## Abstract

Very large unidentified elongate and rounded fossil bone segments of uncertain origin recovered from different Rhaetian (Late Triassic) fossil localities across Europe have been puzzling the paleontological community since the second half of the 19th century. Different hypotheses have been proposed regarding the nature of these fossils: (1) giant amphibian bones, (2) dinosaurian or other archosaurian long bone shafts, and (3) giant ichthyosaurian jaw bone segments. We call the latter proposal the ‘Giant Ichthyosaur Hypothesis’ and test it using bone histology. In presumable ichthyosaur specimens from SW England (Lilstock), France (Autun), and indeterminate cortical fragments from Germany (Bonenburg), we found a combination of shared histological features in the periosteal cortex: an unusual woven-parallel complex of strictly longitudinal primary osteons set in a novel woven-fibered matrix type with intrinsic coarse collagen fibers (IFM), and a distinctive pattern of Haversian substitution in which secondary osteons often form within primary ones. The splenial and surangular of the holotype of the giant ichthyosaur *Shastasaurus sikanniensis* from Canada were sampled for comparison. The results of the sampling indicate a common osteohistology with the European specimens. A broad histological comparison is provided to reject alternative taxonomic affinities aside from ichthyosaurs of the very large bone segment. Most importantly, we highlight the occurrence of shared peculiar osteogenic processes in Late Triassic giant ichthyosaurs, reflecting special ossification strategies enabling fast growth and achievement of giant size and/or related to biomechanical properties akin to ossified tendons.

## Introduction

The Late Triassic covers an extremely long-time span (approximately 36 Ma), encompassing two of the fundamental biological revolutions of interest to paleontology, *i.e.,* part of the Mesozoic Marine Revolution and the End-Triassic Mass Extinction (Harper, 2006; Davies et al., 2017). The Late Triassic also saw the rise of many tetrapod clades in the sea and on land that were to dominate the rest of the Mesozoic (*e.g.*, plesiosaurs and non-avian dinosaurs) or are still prominent today (*e.g.*, mammals). Nonetheless, the complex of biotic interactions of this Mesozoic Epoch and its protagonists still needs to be fully understood (Benton, 2015; Kelley & Pyenson, 2015). Giant ichthyosaurs (length >12 m), prominent elements of the ecological communities of Triassic seas, are no exception due to the absence of satisfactory fossils to unravel their evolutionary history and the still obscure timing, dynamics, and causes of their extinction at the end of the Triassic Period (Lomax et al., 2018; Sander et al., 2021).

### Bone segments and putative giant ichthyosaurs from Europe

Large, but fragmentary bone finds from the famous Aust Cliff Rhaetic bone beds of the Bristol area (southwestern UK) were already reported in the 19th century (Stutchbury, 1850). These include what appeared to be large limb bone shafts of reptilian affinity, leading to extensive discussions in the paleontological community (Stutchbury, 1850; Sanders, 1876; Huene, 1912; Storrs, 1993; Storrs, 1994; Benton & Spencer, 1995; Galton, 2005; Naish & Martill, 2008; Redelstorff, Sander & Galton, 2014; Lomax et al., 2018). The Aust Cliff bone bed is one of a group of similar UK and continental European bone bed-type deposits formed in the Rhaetian epicontinental sea that covered much of Western and Central Europe (Sander et al., 2016; Barth et al., 2018; Cross et al., 2018; Perillo & Heijne, 2023) (Fig. S1). These bone beds yield various tetrapod fossils of both terrestrial and marine origin, often showing fragmentary preservation (Storrs, 1993; Storrs, 1994). The proposed taxonomic affinities of the large to gigantic bone shafts, hereafter less suggestively called “bone segments”, include “labyrinthodonts” (Stutchbury, 1850), dinosaurs (Sanders, 1876; Reynolds, 1946; Storrs, 1993; Storrs, 1994; Benton & Spencer, 1995; Galton, 2005) and unidentified archosaurs (Redelstorff, Sander & Galton, 2014).

The dinosaurian origin of said bone segments (hereafter ‘Dinosaur Hypothesis’) has been supported for the last decades, with Galton (2005) discussing five of the bone segments in detail and concluding that they either must represent sauropodomorph or, more likely, stegosaur long bone shaft fragments (femur, ?tibia). An inconsistency with the long bone nature of the segments would seem to be their lack of a continuous cortex and periosteal surface around their periphery. Instead, as much as two thirds of the periphery of shaft cross sections appears to consist of cancellous bone (Galton, 2005, figs. 4–6). Galton (2005) had already noticed the lack of an outer bone surface in some areas. Whereas this feature could be primary, as in a jaw bone (representing a suture surface or a surface facing the Meckelian canal), it also could result from heavy abrasion, which characterizes all Aust Cliff and other bone bed material.

Galton’s (2005) conclusion as to the stegosaurian nature of the bone segments has since been questioned by multiple workers (Maidment et al., 2008; Naish & Martill, 2008; Sander, 2013; Redelstorff, Sander & Galton, 2014; Lomax et al., 2018) due to the lack of diagnostic morphological features and stratigraphic arguments. In particular, the largest known stegosaur already occurring in the Late Triassic would be inconsistent with the known ornithischian fossil record and result in long ghost lineages (Galton, 2005; Maidment et al., 2008; Naish & Martill, 2008). Sauropods, on the other hand, would appear to be a reasonable option.

A histological test of sauropod affinities of the Aust Cliff bone segments was then conducted by Redelstorff, Sander & Galton (2014). Sampling two of the Aust Cliff specimens (BRSMG-Cb-3869 and BRSMG-Cb-3870, see Table 1) (Redelstorff, Sander & Galton, 2014) found a peculiar and previously undescribed set of histological characters (a thin cortex of fibrolamellar bone with longitudinal primary osteons and secondary osteons forming within the primary ones), inconsistent with sauropod or other sauropodomorph affinities (Redelstorff, Sander & Galton, 2014). In their primary cortex, sauropodomorph long bones show a different and rather uniform histology: laminar and plexiform fibrolamellar bone and, in the case of sauropods, almost no growth marks until late in life (Sander & Klein, 2005; Klein & Sander, 2007; Klein & Sander, 2008; Sander et al., 2011).

**Table 1:** List of specimens used in this study.

Spec. No.	Locality	Age	Strat. Unit	Anatomy	Taxon	Reference	Samples	Sampling method	Plane of section	Thin section repository	Remarks
RTMP-1994-378-0002	Sikanni Chief River, British Columbia, Canada	middle Norian	Pardonet Formation	surangular, splenial	*S. sikanniensis*	Nicholls & Manabe (2004)	2	cut	cross	IGPB	holotype
BRSMG-Cg-2488 R-101	Lilstock, UK	Rhaetian	Top of Westbury Mudstone Formation	surangular	Shastasauridae indet.	Lomax et al. (2018)	1	core	cross	BRSMG	
BRSMG-Cb-3869, 3870, 4063	Aust Cliff, UK	Rhaetian	Rhaetic bone bed at base of Westbury Mudstone Formation	surangular	Shastasauridae indet.	Galton (2005), Redelstorff, Sander & Galton (2014), Lomax et al. (2018)	3	core	cross	BRSMG	
PLV-1964	Autun, France	Rhaetian	Grès à Avicula contorta, Grès Blonds Formation	surangular	Shastasauridae indet.	Fischer et al. (2014), Lomax et al. (2018)	2	core, cut	cross and long	IGPB	
WMNM P-uncatalogued	Bonenburg, Germany	late middle Rhaetian	Exter Formation	cortical fragment	Tetrapoda indet.	Sander et al. (2016)	1	cut	cross	IGPB	
WMNM P88130,..,P88144	Bonenburg, Germany	late middle Rhaetian	Exter Formation	15 cortical fragments	Tetrapoda indet.	Sander et al. (2016)	14	cut	cross and long	IGPB	

Following the recent find of a very large elongate and partially curved bone segment (BRSMG-Cg-2488, 96 cm long, Fig. S3B) in the Rhaetian of Lilstock (Lomax et al., 2018), also in SW England, this segment and the Aust Cliff bone segments were identified as fragments of the surangular bone derived from giant ichthyosaur jaws by Lomax et al. (2018). This interpretation by Lomax et al. (2018) was based on a morphological comparison with somewhat older giant ichthyosaurs from North America, specifically the Carnian *Shonisaurus popularis* from Nevada (Camp, 1980) and the Norian *Shastasaurus sikanniensis* (Fig. S3A) from British Columbia, Canada (Nicholls & Manabe, 2004). We term this hypothesis of the affinity of the very large Aust Cliff bone segments the ‘Giant Ichthyosaur Hypothesis’.

Support for the Giant Ichthyosaur Hypothesis would seem to come from an earlier find, now lost (Fig. S3C). Huene (1912) described a 1.4 m long bone segment from Aust Cliff which he identified as the fragment of a right lower jaw of a giant ichthyosaur, including part of four elements (dentary, splenial, angular, surangular) (Fig. S3C). Huene (1912) noted that this fossil had been accessioned to the “Bristol Museum” since 1877, presumably referring to today’s Bristol City Museum and Art Gallery (BRSMG). However, Huene (1912) did not provide a specimen number, and since his 1912 study, the specimen has not been mentioned again, and it may well have been destroyed in WWII. According to Huene’s (1912) description and illustration, the specimen consists of four non-fitting parts, the penultimate of which had been sectioned transversely (Fig. S3C) at some earlier point in time before Huene’s study.

Curiously, among the putative dinosaur long bone material described by Galton (2005) from Aust Cliff, there also is a transversely sectioned specimen (BRSMG-Cb-3870, Fig. S2) of about the dimensions noted by Huene (1912) (Fig. S3C). Galton did not cite Huene, and there is a possibility that the two authors did study the same specimen. Arguing against the identity of the two specimens is the poor preservation of the Galton specimen (whereas Huene emphasized the good preservation of his material) and the fit with another segment (whereas Huene noted the lack of fits).

Finds similar to the Aust Cliff and Lilstock material have come from the epicontinental French Rhaetian localities of the Autun area (Fig. S1) and from southern France (Fischer et al., 2014; Lomax et al., 2018), as well as most recently, from the German locality of Bonenburg (Fig. S1) (Sander et al., 2016; Wintrich et al., 2017) and the Swiss Alps (Sander et al., 2022, fig. s5). Fischer et al. (2014) also had interpreted their material as ichthyosaurian but did not extend their considerations to the UK material and did not cite Huene (1912). Huene (1912), on the other hand, just described this one specimen from Aust Cliff and did not comment on the putative dinosaur leg bone shafts from the same locality nor on the French Rhaetian ichthyosaur material, all of which were known at the time.

### The Late Triassic giant ichthyosaur record

Ever since the work of Charles S. Camp on *Shonisaurus popularis* from Berlin Ichthyosaur State Park in the Carnian Luning Formation of Nevada, USA, in the 1950s (Camp, 1980), it has been clear that Late Triassic ichthyosaurs reached body lengths of 15 m or more and must have been substantially larger than post-Triassic ichthyosaurs. The *S. popularis* material has been reevaluated several times since with regard to its size, skeletal reconstruction, taphonomy, and reproductive biology (Kosch, 1990; Hogler, 1992; McGowan & Motani, 1999; Kelley et al., 2022). Even larger and more complete than any of the *S. popularis* finds is the holotype skeleton of *Shastasaurus sikanniensis* (Nicholls & Manabe, 2004) from the middle Norian of British Columbia, Canada. Based on field data, this individual is estimated to have been 21 m long (Nicholls & Manabe, 2004).

It is also now acknowledged that various other ichthyosaur finds from the Late Triassic must represent animals over 10 meter in length, but most giant ichthyosaurs are represented by woefully incomplete, disarticulated, and fragmentary material from around the world (Callaway & Massare, 1989; McGowan & Motani, 1999; Sander et al., 2022; Kelley et al., 2022) which hinders the anatomical descriptive effort. In continental Europe, the fragmentary, often reworked, and poorly understood finds attributed to giant ichthyosaurs come from late Norian to Rhaetian outcrops of France (Fischer et al., 2014), the eastern Swiss Alps (Sander et al., 2022), and from a recently discovered Aust Cliff-type bone bed near the central German village of Bonenburg (Fig. S1) (Sander et al., 2016; Wintrich et al., 2017). Unlike all the other Rhaetian localities with putative giant ichthyosaurs, the Bonenburg deposit is precisely dated palynologically, ranging from late middle to early late Rhaetian in age (Schobben et al., 2019; Gravendyck et al., 2020). The Bonenburg ichthyosaur fossils include large but very short vertebral centra, a very large neural arch, and very large rib fragments (Sander et al., 2016)). In addition, the bone bed frequently yields heavily abraded fragments of thick cortical bone up to 25 cm in length (Figs. S4A, S6A), which we hypothesize to be fragments of bone segments similar to the more complete British and French specimens (Figs. S2A, S3B).

Understanding the affinity of the fragmentary Late Triassic ichthyosaurs and of the large, more obscure fragmentary finds, is important because of the absolute size of these remains, representing records of the largest animals inhabiting the Late Triassic oceans (Lomax et al., 2018; Sander et al., 2022). The fossils represent animals that far exceeded the size of any other marine tetrapods except for the largest species of baleen whales and archaeocetes (Bianucci et al., 2023). The importance of these fossils also relates to the patterns of extinction at the end of the Triassic, given that very large ichthyosaurs appear to have persisted to the late Rhaetian (indicated by the Bonenburg finds) but are lacking in the Jurassic.

The lack of clear and unequivocal external morphological features in the Rhaetian European bone segments due to their fragmentary and reworked nature makes alternative approaches such as microstructure analysis (microanatomy and osteohistology) critically important for investigating the possible affinities of these fossils. Both Galton (2005) and Lomax et al. (2018) illustrated cross sections of UK fossils and discussed microanatomy (but not histology, which is not accessible without thin-sectioning). Galton compared the midshaft microanatomy of BRSMG-Cb-3869, 3870, and 4063 from Aust Cliff to that of various dinosaurs and concluded that the fossils must represent stegosaurs based on the coarse cancellous bone structure of the medullary region. Lomax et al. (2018) noted and illustrated in detail the same coarse cancellous bone structure but did not use microanatomical arguments as evidence for determining affinity, only cross-sectional shape. Histological analysis was already performed on two Aust Cliff specimens (BRSMG-Cb-3869 and BRSMG-Cb-3870) by Redelstorff, Sander & Galton (2014) (Table 1), but without considering possible ichthyosaurian affinities of the fossils.

Here we undertake a detailed and comprehensive comparison and sampling of most European Rhaetian “bone segments” and putative giant ichthyosaur jaws for histological analysis. The main aim of this study thus is to histologically test the Giant Ichthyosaur Hypothesis by searching for shared histological characters among European material of confirmed or proposed ichthyosaurian nature, on one hand, and bonafide Late Triassic giant ichthyosaurs, such as *S. sikanniensis*, on the other. We also compare the “bone segments” histology with other terrestrial and aquatic tetrapods that are known to have reached very large body size in the Late Triassic such as sauropodomorph dinosaurs, rauisuchians, dicynodonts, and plesiosaurs.

## Materials & Methods

### Materials

The material used in this study consists of bone histological samples taken from various specimens borrowed from multiple institutions as listed in Table 1. Abbreviations for these institutions are also listed in this table. In summary, there are eight sets of samples (Table 1). These include two samples (surangular, splenial) from the *S. sikanniensis* holotype RTMP-1994-378-0002 (Nicholls & Manabe, 2004) (Fig. S3A), one sample of the Lilstock putative ichthyosaur surangular (Lomax et al., 2018) (Figs. S2, S3B), three samples of “dinosaur bone shafts” reinterpreted as giant ichthyosaur jaw bone fragments from the Aust Cliff Rhaetic bone bed (Galton, 2005; Redelstorff, Sander & Galton, 2014; Lomax et al., 2018), two samples from a giant putative ichthyosaurian lower jaw (Fischer et al., 2014), identified as surangular by Lomax et al. (2018), from Autun, France (Figs. S2, S3B), and finally 16 cortex fragments of various sizes from Bonenburg, Germany (Figs. S4, S5A, S6A). For details on all of these samples, including sampling locations and methods, and their current identification, see Article S1.

The thin sections used for the study are either in the paleohistological collections of the IGPB or with the sampled fossils (see Table 1). Note that two of the Aust Cliff thin sections were already studied by Redelstorff, Sander & Galton (2014).

### Methods

#### Histological sampling

Except for the *S. sikanniensis* holotype, jaw bones and putative jaw bones were sampled by core drilling, following Sander (2000) and Stein & Sander (2009) (Table 1). The *S. sikanniensis* lower jaw was sampled with a Dremel-type cutting tool, making two parallel cuts spaced 18 mm apart (Fig. S3E) and then preparing out the sample. Complete cross sections and longitudinal sections were obtained from smaller specimens of cortical bone fragments from Bonenburg by cutting with a rock saw after embedding with a protective epoxy putty. Cores and full sections were then processed into thin sections following Lamm (2013), with slight modification of the standard technique: wet silicon carbide powder of grit sizes of 600 and 800 was used for the grinding and polishing processes.

Once covered, the thin sections were studied under a Leica DMLP polarizing light microscope in regular illumination and by using cross-polarization and circular polarization techniques. Circular polarization (Bromage et al., 2003) was obtained through the use of a pair of commercially available polarizing glasses for 3D movie viewing to replace the polarizer and the analyzer of the microscope (Richtberg & Girwidz, 2017). This allows observation of the thin sections in circular polarized light without the Maltese cross effect. Photomicrographs were taken using a Leica DFC420 camera (software Leica Firecam, ver. 3.1, 2007, ©Leica Microsystems, Switzerland, Ltd), a Dino-Eye camera (software DinoCapture 2.0 ver 1.5.45 ©2016 AnMo Electronics Corporation), and with a Canon EOS2000D (software EOS Utility ver. 3.16.11, 2023, ©Canon Europa N.V. and Canon Europe Ltd 2002–2009) mounted on the microscope.

#### Porosity quantification

The thin sections prepared from the samples (see above) were scanned with a flatbed scanner or photographed under the microscope with a cell phone camera. In the latter case, successive microphotos were merged using the photomerge tool in Photoshop (Ver 20.0.4 20190227.r.76). Both scans and merged photos were transformed into binary pictures for porosity quantification (Fig. S7). Porosity quantification was executed with the software BW-counter (©Peter Göddertz, IGPB). Porosity is expressed as the percentage of white area (vascular and trabecular cavities) *vs.* black area (mineralized bone material). To assess the porosity of different areas of a thin section (*e.g.*, outer cortex *vs.* deep cortex), the binary pictures were hand cropped according to the subdivisions of the cortex described in the Results section.

#### Terminology, including new terminology

Histological terminology follows Buffrénil & Quilhac (2021a) for osteohistology and Buffrénil & Quilhac (2021b) for types and features of secondary osteons. These play a major role in this study. For one, there are concentric osteons, most recently discussed by Buffrénil & Quilhac (2021b). In these, a secondary osteon develops within a Haversian canal, *i.e.,* within a preexisting secondary (not primary) osteon (Lacroix, 1970). We did observe concentric osteons in this study. Concentric osteons are not to be confused with double-zoned secondary osteons (Skedros, Sorenson & Jenson, 2007) where the centripetal infill of a secondary osteon happens in stages, but without intervening resorption. We did not observe such double-zoned secondary osteons in this study. However, neither of these terms describes the situation observed already by Redelstorff, Sander & Galton (2014) in the Aust Cliff material, in which a secondary osteon develops within a primary one. We refrain from erecting new terminology for this situation but use a simple descriptive approach. When the entire cortex is affected by the reuse of preexisting vascular canals by secondary osteons, we define this as “template cortex”.

Nevertheless, the histology of the giant ichthyosaur material is so unusual in other features that it does require new terminology which will be introduced in the results section. This new terminology was coined to aid in the description of a novel histology in the periosteal territory for which no proper definition was found in the literature.

Our general histological description follows the 3-Front Model of Mitchell & Sander (2014) in which the osteohistological pattern observed in an amniote cortical bone sample is conceptualized as being generated by the successive outward advance and relative speed of three fronts. Due to the undefined taxonomical state of the specimens and lack of clear homology in sampling location (aside for BRSMG-Cb-3869 and BRSMG-Cg-2488 R-101), the model is only used for descriptive purposes and general comparison, but not to define relative developmental stages.

## Results

### Shared histology of the British and French samples

#### General histological and microanatomical description

**Figure 1:**
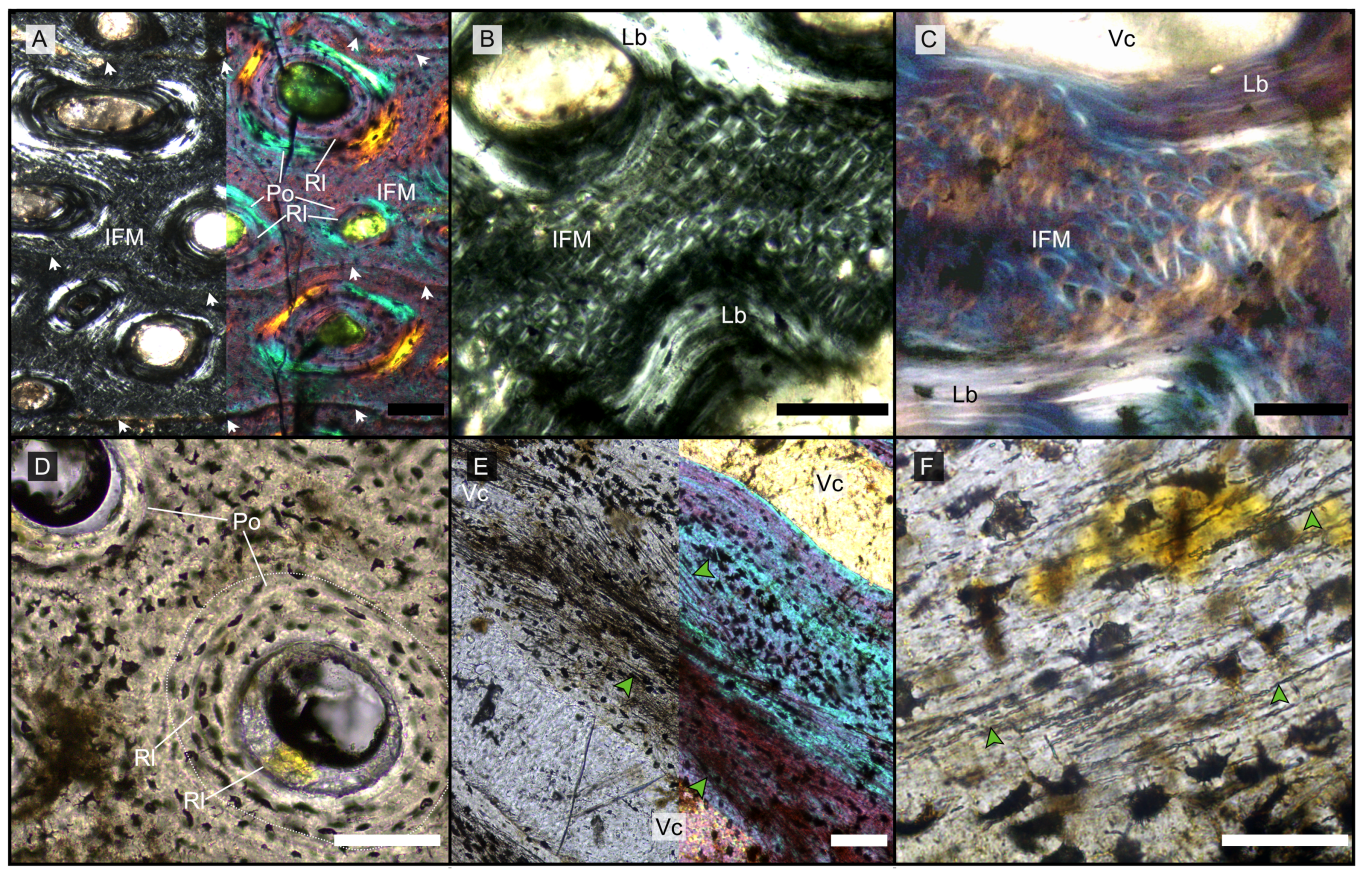
Main histological features of the giant ichthyosaurs lower jaws. (A) BRSMG-Cg-2488 R-101 seen in cross-polarized light (left) and with a lambda filter added (right). The specimen shows a regular arrangement of rows of primary osteons with secondary osteons within, separated by thin periosteal GM (white arrows), and a high number of osteocyte lacunae. (B) Polarized light view of BRSMG-Cg-2488 R-101 showing the grid pattern of periosteal intrinsic fibers that characterizes the intrinsic fiber matrix (IFM). (C) BRSMG-Cg-2488 R-101 in circular polarized light revealing the seemingly helicoidal arrangement of the periosteal structural fibers and their interconnection within osteonal lamellar bone (top left). (D) Normal light view of the cross section of PLV-1964 showing two primary osteons. The right one (dotted line) shows a secondary osteon within the primary one. (E) Longitudinal section of PLV-1964 showing strands of unmineralized fibers (dark) running longitudinally in a herringbone pattern (green arrows) in normal light (left) and in polarized light with lambda filter (right). (F) PLV-1964 in normal light showing the irregular shape of osteocyte lacunae and the unmineralized fibers (green arrows). *Abbreviations*: Lb, lamellar bone; Po, primary osteon, IFM, intrinsic fiber matrix; Rl, resorption line; Vc, vascular canal. Scale bars equal 100 µm (A, B, D, E), and 50 µm (C, F).

**Figure 2:**
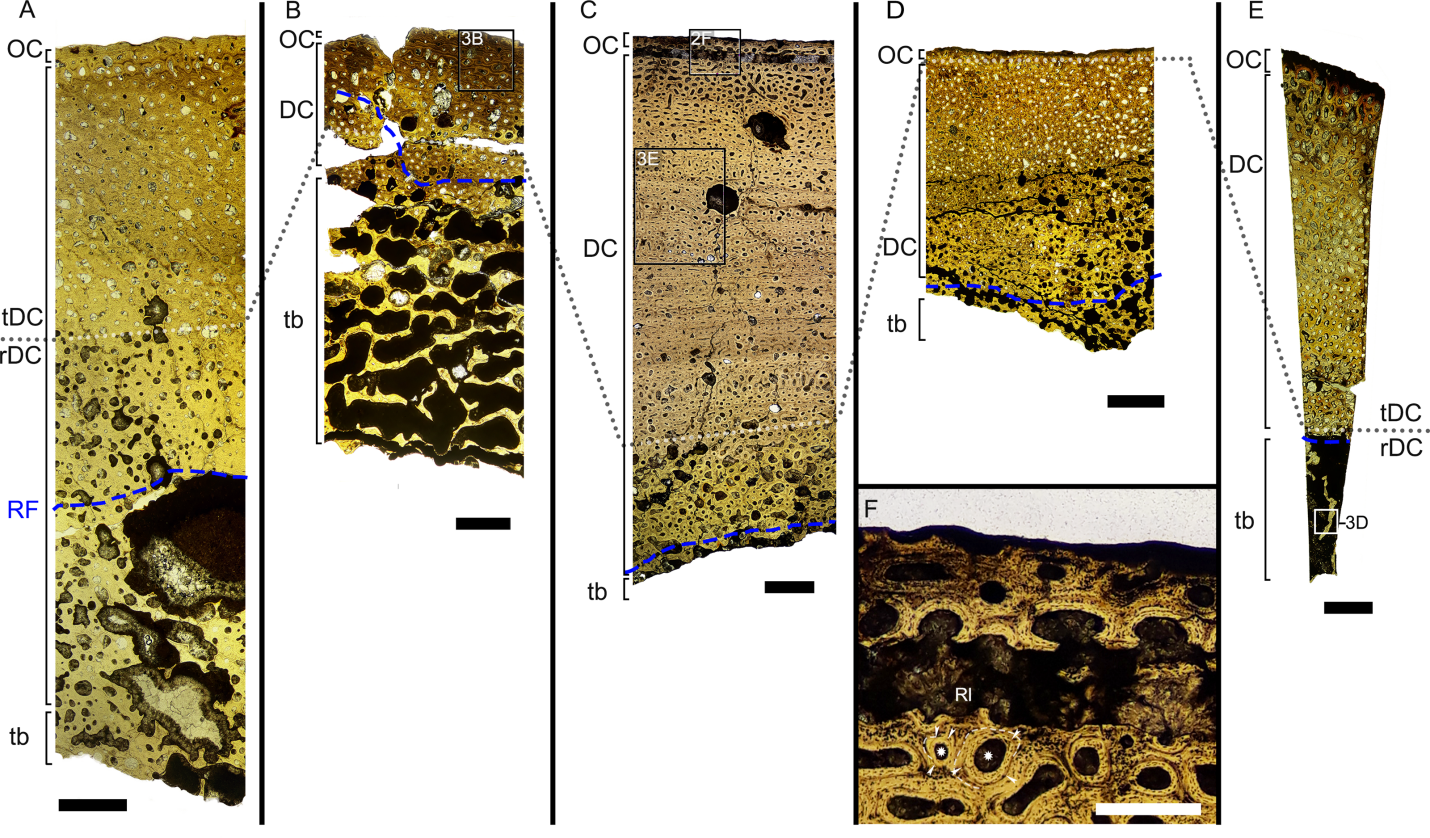
Overview of composite micrographs of selected thin sections. The resorption front is indicated by a blue dashed line, a black dotted line indicates the boundary between rDC and tDC. (A) BRSMG-Cb-3869, from Aust Cliff; (B) BRSMG-Cb-3870, from Aust Cliff; (C) BRSMG-Cg- 2488, from Lilstock; (D) BRSMG-Cb-4063, from Aust Cliff; (E) PLV-1964, from Cuers; (F) detail of the outer cortex of BRSMG-Cg- 2488 showing secondary osteons (white stars). White dotted lines indicate the still visible borders of primary osteons, white arrowheads indicate the resorption lines of the secondary osteons. Abbreviations: DC, deep cortex; OC, outer cortex; rDC, regular deep cortex; RF, resorption front; So, secondary osteon; tb, trabecular bone; tDC, template deep cortex. Scale bars equal 2 mm (A–E), and 500 µm (F).

**Figure 3:**
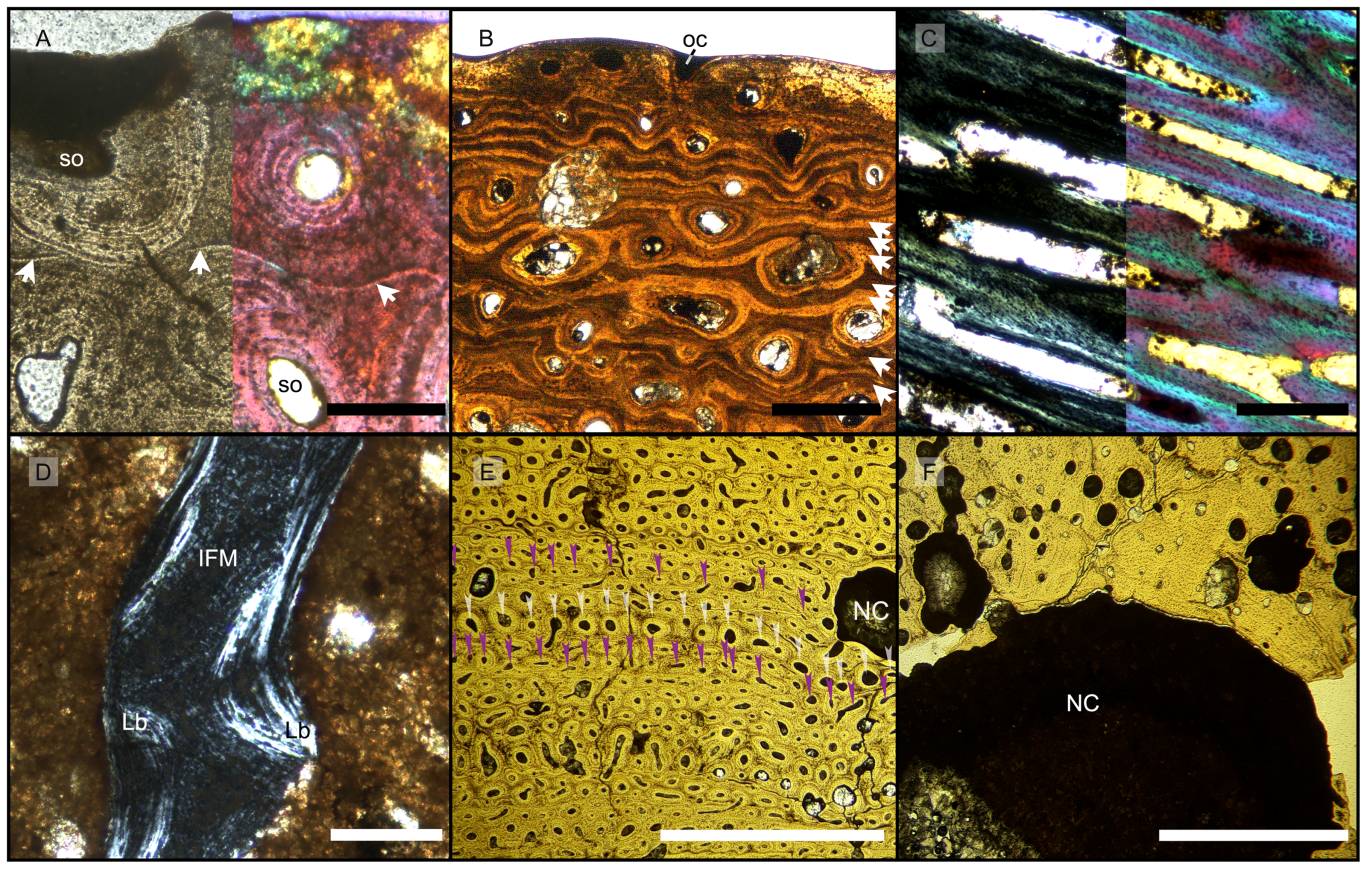
Features characterizing the areas identified as outer cortex, trabecular bone and deep cortex. (A) Outer cortex of BRSMG-Cb-4063 in normal light (left) and in cross-polarized light with lambda filter added (right) showing primary tissue and growth marks (white arrows). Secondary osteons are present on the outer edge of the bone and may interrupt the continuity of the GM. The outer surface also shows diagenetic damage leading to the opening up of a secondary osteon. (B) BRSMG-Cb-3870 showing GM (white arrows) and a vascular canal open to the outer bone surface. (C) Longitudinal section of PLV-1964 in cross-polarized light (left) and with a lambda filter added (right) revealing longitudinal vascularization. (D) Detail of trabecular bone of PLV-1964 showing primary IFM and secondary lamellar bone in cross-polarized light. (E) BRSMG-Cg-2488 R-101 showing a template cortex characterized by parallel rows of primary and secondary osteons (white and purple narrow arrows) bordered by successive GM. Note the steep downturning of the rows in the vicinity of the nutrient canal. (F) Nutrient canal of BRSMG-Cb-3869 in normal light showing the presence of primary simple vascular canals and resorption cavities on the outer edge of the canal. *Abbreviations*: Lb, lamellar bone; NC, nutrient canal; oc, open vascular canal; IFM, intrinsic fiber matrix; so, secondary osteon. Scale bars equal 100 µm (A, C, D), 500 µm (B), and 2 mm (E, F).

Laid down by the apposition front (Mitchell & Sander, 2014), the outer cortex of all samples from the British and French Rhaetian is characterized by compact primary bone tissue structured by wavy growth marks parallel to the outer bone surface (Figs. 1A, 2, 3A, 3B).

The primary periosteal bone matrix is a new matrix type, “intrinsic fiber matrix” or IFM. IFM is characterized by a network of bright anisotropic, intrinsic, mineralized fibers set in an isotropic matrix. IFM pertains to the woven-fibered type of bone matrices which are produced by static osteogenesis (Buffrénil & Quilhac, 2021a). It is generally accepted that the degree of organization of the collagen fibers in a bone matrix is negatively correlated with its rate of deposition (Buffrénil & Quilhac, 2021b). Because of its structure being more organized than simple woven bone but less organized than parallel fibered bone, IFM probably represents the result of an intermediate type of bone deposition between these two bone matrix types. Contrary to a normal woven-fibered bone matrix, IFM contains abundant coarse intrinsic collagen fibers, both mineralized and unmineralized, that are uniformly oriented longitudinally. Contrary to parallel fibred bone, IFM coarse fibers have different orientation showing a net or lattice-like pattern, immersed in a clearly isotropic matrix. Of particular relevance is that the fibers are intrinsic, not extrinsic (as *e.g.*, Sharpey’s fibers).

Vascularization of the primary cortex is characterized by longitudinal vascular canals (Figs. 2, 3A, 3B). Immature primary osteons and vascular canals open up to the outer bone surface, resulting in an ornamented wavy surface (Figs. 2, 3B) in thin section. This histology correlates with distinctive longitudinal surface striations on the specimens (Fig. S3D), nicely illustrated by Lomax et al. (2018, fig. 4c, 8) for the Lilstock and Aust specimens and Fischer et al. (2014; fig. 2, s5) for the French specimens.

Vascular canals and primary osteons are arranged in appositional circumferential rows demarcated by closely spaced growth marks (GM) that vary in number (Figs. 1A, 2, 3A, 3B). Growth marks appear as depositional layers of periosteal primary bone and run around the periosteal vascular canals (Figs. 1A, 3A, 3B), appearing to embrace them. The GM vary in thickness (Figs. 1A, 3A *vs* 3B) and show alternating light-dark coloration. The differential coloration seems to be related to differences in intrinsic fiber density and orientation. Vascularization as observed in longitudinal sections does not show anastomoses between vessels, with vessel cross sections rarely showing shapes more complex than an elongated ellipsoid (Fig. 3C).

The Haversian substitution front is diffuse in that the outer cortex shows scattered evidence of secondary remodeling through small resorption cavities and secondary osteons within primary ones (Figs. 1A, 1D), or even mature secondary osteons (Fig. 3A). Appositional rows of primary osteons may follow or precede rows of primary osteons with secondary ones within them, or rows of secondary osteons may even be intercalated with rows of purely primary osteons (Figs. 1A, 1D, 3E). The deep cortex, *i.e.,* the part of the cortex that is fully within the Haversian substitution front, can again be subdivided in an outer template deep cortex and an inner, completely remodeled area, where none of the primary pattern of vascularity is preserved (Fig. 2). This situation was already described in detail by Redelstorff, Sander & Galton (2014, fig. 4). The thickness of these two subzones of the deep cortex varies between samples (Fig. 2).

As noted above, the template deep cortex is so named because it preserves most or some of the original primary vascular architecture (Figs. 2A–2E, 3E). This is because of the peculiar pattern that Haversian substitution is initiated from existing primary vascular canals, *i.e.,* existing vascular pathways were reused. This results in a predominance of secondary osteons within primary ones. Of further relevance is the generally small diameter of the secondary osteons, which is comparable to the diameter of primary osteons (Figs. 1A, 1D, 2F, 3A, 3AE), unlike what seen in other amniote taxa (see Discussion). The primary osteons thus clearly influence the course of the secondary ones, even leading to rows of exclusively secondary osteons forming complete Haversian tissue, templated by the primary rows of osteons (Fig. 3E). The templating we observed is different from normal Haversian substitution in amniotes in which the cutting cones of secondary osteons show little regard for preexisting structures (Mitchell, 2017).

Both, primary and secondary osteons, have a high number of lamellae and a small vascular canal, which results in a rather low average porosity for the entirety of the sections (between 17% and 13%) (Figs. S7A–S7E), possibly indicating an osteosclerotic state of the cortex. Porosity generally decreases from the deep cortex toward the outer cortex, except for BRSMG-Cg-2488 R-101, which shows rows of widened vascular canals at the transition between deep and outer cortex.

Osteon cross sections vary consistently between circumferential rows, sometimes horizontally flattened, sometimes more vertically (Figs. 2, 3E), indicating modulations resulting from variations in growth rate (Woodward, 2019). Migratory and incipient osteons (Skedros, Sorenson & Jenson, 2007; Mitchell, 2017) are present, but secondary osteons within primary ones represent the majority of osteons in the template cortex.

Further inward from the template cortex, the regular deep cortex can be seen as resulting from complete secondary reconstruction (Figs. 2A–2D). This part of the cortex is characterized by more chaotically arranged secondary osteons that have obliterated the primary vascular architecture by several cycles of secondary osteon formation. The result is normal Haversian tissue which marks the full effect of the Haversian substitution front (*e.g.*, Fig. 2D).

The boundary of the perimedullary region, *i.e.,* the resorption front, is also diffuse (Figs. 2A–2D). Here, the deep cortex becomes more and more affected by larger resorption cavities lined only by a few lamellae. Porosity in the perimedullary region is between 65 to 85% (Figs. S7A–S7E). This signifies an increasing imbalance between secondary bone deposition and resorption activity and initiates the formation of secondary trabeculae (Figs. 2A–2E). Through the activity of the resorption front, the perimedullary region is rich in resorption cavities replacing bone tissue with some secondary osteons and transitioning to a medullary area of secondary trabecular bone (Figs. 2A–2E).

In cases, where the resorption front has overtaken the Haversian substitution front, interstitial areas of primary tissue consisting of IFM are visible (Fig. 3D) between the secondary trabeculae. The percentage of interstitial primary tissue decreases inwards but is patchy. Our histological observations are consistent with the descriptions and illustrations of cross-sectional microanatomy given by Galton (2005), Fischer et al. (2014), and Lomax et al. (2018), who all note that there is only a very small open medullary cavity surrounded by an extensive zone of inward-decreasing trabecular density (Galton, 2005, figs. 4–6; Fischer et al., 2014, fig. s5; Lomax et al., 2018, fig. 6). Fischer et al. (2014, fig. s5) interpret this open medullary cavity as the dental groove, however.

Both, the largest Aust Cliff bone segments (BRSMG-Cb-3869) and the Lilstock specimen (BRSMG-Cg-2488 R-101), show conspicuous cavities in the cortical bone (Figs. 2A, 2C, 3F). There are two obvious ones in the latter and one obvious and a second possible one in the former (Figs. 2A, 2C, S7A, S7C). Based on the sampling location, these open cavities represent the nutrient canals extending inwards at a low angle from the elongate foramen (Figs. S2A, S3B–S3C) opening in caudal direction (already described by Huene, 1912 and identified as part of the *fossa surangularis* by Lomax et al., 2018) on the bone surface. On the outward margins of the cavities (those facing the periosteal surface), both samples show primary tissue and simple vascular canals (Fig. 3F). Both BRSMG-Cb-3869 and BRSMG-Cg-2488 R-101 show signs of resorption along the inner and lateral margins of the nutrient canals, indicating microanatomical drift related to the growth of the bone enclosing the canal.

#### Template remodeling and secondary osteons within primary ones

As noted, the distinctive template secondary remodeling is shared between all French and UK samples (Table 2). It is possible to identify secondary osteons within primary ones through the method adopted by Redelstorff, Sander & Galton (2014), *i.e.,* focusing through the sample in normal light, using higher magnifications, a nearly closed diaphragm, and the condenser, or by observing the position of the resorption/cementing lines through the *λ* filter. The occurrence of multiple generations of secondary osteons within primary ones, of comparable diameter (Figs. 1A, 1D), tends to maintain the original periosteal appositional rows (tDC in Figs. 2A, 2B, 2C, 2E). Secondary osteons within primary ones represent the advancing Haversian substitution front and may occur quite closely to the outer bone surface in the outermost cortical layers (Fig. 2F). Whereas secondary osteons within primary ones also have been reported in various mammals (*e.g.*, Sander & Andrassy, 2006 and the reference cited above), they are not such a consistent and pervasive feature in these mammals compared to the template cortex of our specimens.

**Table 2:** Comparison of the osteohistological features across our study sample and other Late Triassic taxa from the literature. The results here summarized are based on literature research and, when available, on authors’ observation of the samples in the IGPB histology collection.

Groups considered in this study	Source of histological samples	Main bone organization	Vascularization rate	Vascular organization	Cyclical structures	Periosteal remodeling strategy	Relative remodeling rate	Abbundant concentric osteons	Main references
Lilstock ichthyosaur	Lower jaws	WPC with IFM (PIFT)	High	Longitudinal	GM	Template+diffused	High	Yes	This study
Autun ichthyosaur	Lower jaws	WPC with IFM (PIFT)	High	Longitudinal	GM	Template+diffused	High	Yes	This study
Aust Cliff bone segments	Lower jaws(?)	WPC with IFM (PIFT)	High	Longitudinal	GM	Template+diffused	High	Yes	Redelstorff, Sander & Galton (2014), this study
*S. sikanniensis*	Splenial and surangular	WPC with IFM (PIFT)	High	Longitudinal	Not preserved	Not preserved	Not preserved	Not preserved	This study
Bonenburg cortical fragments	Unidentified cortices	WPC with IFM (PIFT)	High	Longitudinal	GM	Template+diffused	High	Yes	This study
Sauropodomorpha	Long bones	WPC (fibrolamellar)	Moderate to high	Plexiform/laminar	LAGs	Organized front	Moderate to high	Not observed or reported	Klein & Sander (2007), Mitchell & Sander (2014)
Stegosauria	Long bones	WPC	Moderate	Longitudinal	LAGs	Scattered front	Moderate to high	Not reported	Redelstorff & Sander (2009), Padian & Woodward (2021)
Rauisuchia - Slow growth	Long bones	Lamellar-zonal+WPC	Low	Laminar/subplexiform	Annuli+LAGs	Scattered	Low	Not reported	Ricqlés, Padian & Horner (2003), Ricqlés, Buffrénil & Laurin (2021)
Rauisuchia - Fast growth	Long bones	WPC	Moderate to high	Laminar/subplexiform	Annuli	Scattered	Low	Not reported	Klein, Foth & Schoch (2017), Buffrénil, Quilhac & Cubo (2021)
Phytosauria	Long bones	Lamellar-zonal	Low	Longitudinal	Annuli+LAGs	Scattered front	Low	Not reported	Ricqlés, Padian & Horner (2003), Ricqlés, Buffrénil & Laurin (2021)
Dicynodontia	Long bones	WPC	Moderate to high	Longitudinal	GM	Scattered and unorganized		Not reported	Chinsamy & Rubidge (1993), Green, Schweitzer & Lamm (2010)
Plesiosauria	Long bones	WPC	Moderate to high	Longitudinal+radial	GM	Template+front	High	Yes (?)	Wintrich et al. (2017), Sander & Wintrich (2021)
Nothosauria	Ribs	WPC+CPF	Moderate to high	Longitudinal+radial	LAGs	Absence	Low	Not reported	Klein, Canoville & Houssaye (2019)
Temnospondyili	Lower jaws, long bones	Lamellar-zonal+ISFs	Low to moderate	Longitudinal+plexiform	Annuli+LAGs	Template	Low to moderate	Not reported	Konietzko-Meier et al. (2018), Gruntmeijer, Bodzioch & Konietzko-Meier (2021)

**Notes.**

Abbreviations CPF coarse paralle fibered bone GM growth marks IFM intrinsic fiber matrix ISFs interwoven structural fibers LAGs Lines of arrested growth WPC woven-parallel complex PIFT periosteal intrinsic fiber tissue

#### PIFT with longitudinal vascular canals

All UK and French Rhaetian putative jaw bone samples share the same unusual primary periosteal bone tissue: a woven-parallel complex with strictly longitudinal, highly ordered, primary osteons set in intrinsic fiber matrix (IFM). We term this woven-parallel complex ‘periosteal intrinsic fiber tissue’ PIFT (Table 2). PIFT is a feature at the bone tissue level of integration (covering the different types of bone tissues) and thus is to be used in conjunction with ‘lamellar bone tissue’, ‘parallel-fibered tissue’, ‘Haversian tissue’, *etc.* (Buffrénil & Quilhac, 2021a, Buffrénil & Quilhac, 2021b). In PIFT, the “parallel” component of the woven-parallel complex is represented by typical osteon lamellar bone of the longitudinal primary osteons, the “woven” component, building up the scaffold of the bone, is IFM (Figs. 1A–1C, S8F). IFM is a type of woven-fibered matrix (Stein & Prondvai, 2014, Buffrénil & Quilhac, 2021a) because it is a combination of isotropic woven bone with coarse intrinsic collagen fibers observable in cross (*e.g.*, Fig. 1B) and longitudinal section (*e.g.*, Fig. 1E).

In cross sections under cross-polarized light, IFM is easily identifiable by the presence of a networks of intrinsic fibers, birefringent in cross sections (Fig. 1B), conspicuous against the dark matrix of woven bone (Fig. 1B). The width and length of the intrinsic fibers is variable, and strands intertwine with each other, overlapping in a fabric-weave pattern (Figs. 1A–1C). Circular polarization reveals the true arrangement of these fibers to be circular and coiled (Fig. 1C). The rectangular and hexagonal shape seen with crossed polarizers is thus revealed to be an artifact resulting from the Maltese cross effect. The IFM shows heterogeneity in brightness. The areas of denser fibers often correlate with lower brightness under cross-polarized light in both longitudinal and transverse sections (Fig. 1E).

In longitudinal section, IFM is characterized by bundles of short parallel fibers that intertwine at various angles, from acute to orthogonal (Fig. 1E). These extend across the surface paralleling the direction and angles of the vascular canals (Fig. 1E). The fibers appear as black strands in the tissue and show no birefringence, similar to osteocyte lacunae and canaliculi, indicating a non-mineralized state of these structures (*e.g.*, Wolf, Kalthoff & Sander, 2012).

Osteocyte lacunae are extremely numerous in the IFM and show a wide variety of shapes, from irregularly plump to discoid flattened (Figs. 1D, 1F). The distribution of osteocyte lacunae is generally irregular with no apparent relationship to other histologic features. Lacunae are very dense in some areas and almost absent in others. These dense irregular osteocyte lacunae are left by multipolar static osteocytes, as is typical of a woven-fibered matrix (Stein & Prondvai, 2014; Buffrénil & Quilhac, 2021a). Osteocytes tend to form chaotic clusters where strands and bundles of non-mineralized fibers are present (Fig. 1E). Given the variability in shape and size of both the lacunae and their canaliculi (which are sometimes visible, sometimes not), the more spindle-shaped osteocyte lacunae found in the IFM may represent fibrocytes. The number of osteocyte lacunae is also high in the primary osteons, with a centripetal density increase.

### Histology of indeterminate cortical fragments from Bonenburg, Germany

**Figure 4:**
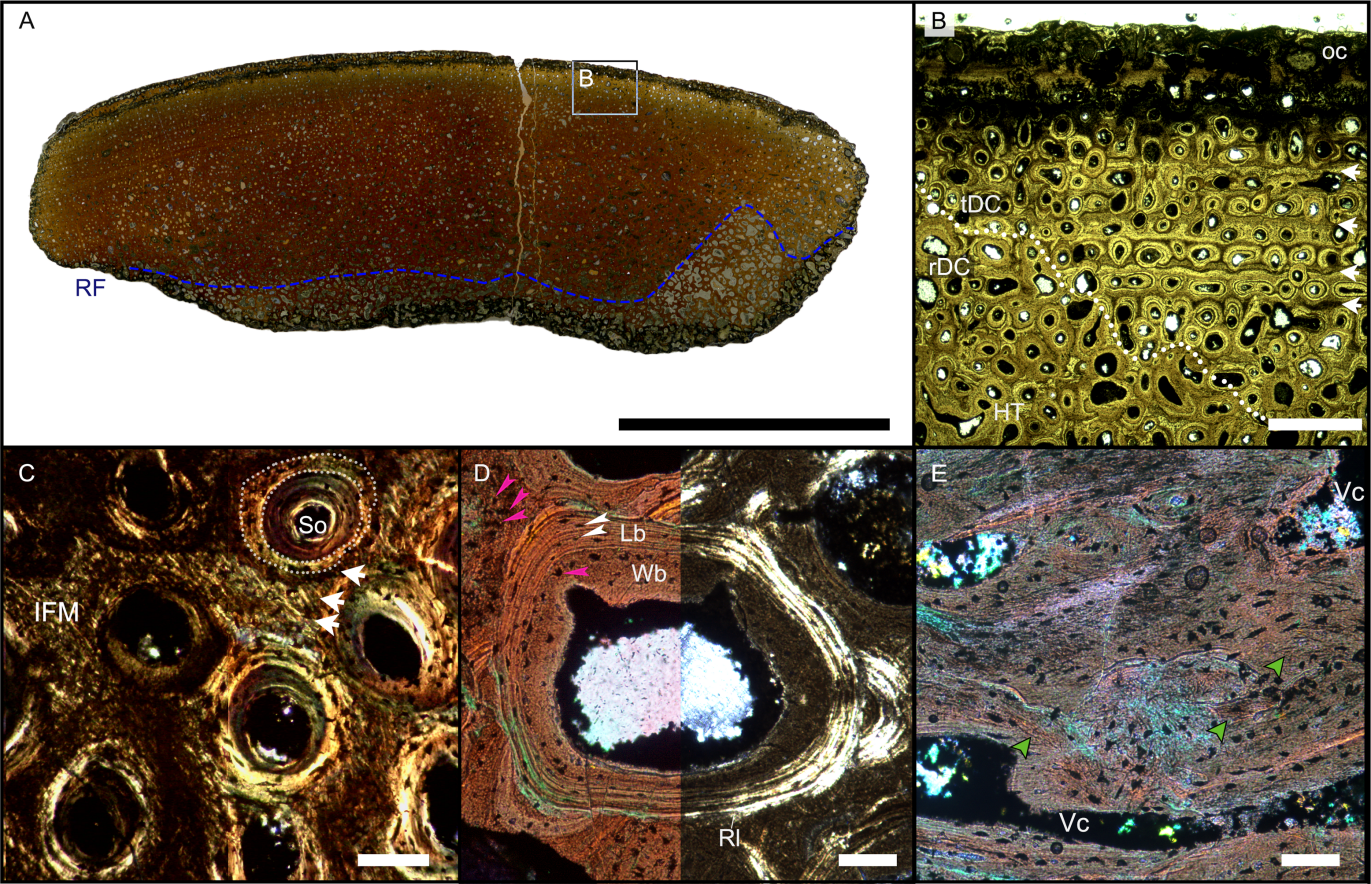
Overview of WMNMP88133, the largest cortical bone fragment from the late Middle Rhaetian of Bonenburg, Germany. (A) Cross section showing a dark diagenetic seam staining the outer bone surface and the resorption front (blue dotted line). Note the low curvature of the outer bone surface and the great thickness of the cortex, suggesting that the fragment derives from a very large bone. (B) Overview of the external cortex showing the characteristic, strictly longitudinal vascular canals arranged in circumferential rows, vascular canals open to the outer bone surface (partially hidden by the dark seam), secondary osteons inside primary ones, and concentric secondary osteons. The obliteration of the multiple parallel rows of GM (white arrows) reveals the border between rDC and tDC (white dotted line). (C) Detail of the tDC, showing secondary osteons and IFM (left half of image cross-crossed polarized light, right half circular polarized light). The intrinsic fibers form parallel GM of alternating colors (white arrows). (D) Secondary osteon filled in by lamellar bone followed by woven or poorly mineralized bone (left side cross-polarized light with lambda filter, right side cross-polarized light only). Pink arrowheads point at the numerous plump or irregularly shaped osteocyte lacunae in the IFM and in the innermost layer of the osteon. White arrowheads points to the less numerous flattened osteocyte lacunae (white arrows) in the lamellar bone. (E) Longitudinal section seen in cross-polarized light with a lambda filter showing unmineralized fiber strands (dark, green arrows). *Abbreviations*: Cl, cementing line; HT Haversian tissue; IFM, intrinsic fiber matrix; Lb, lamellar bone; oc: open vascular canal; rDC, regular deep cortex; RF, resorption front; tDC, templating deep cortex; So: secondary osteon; Vc, vascular canal; Wb, woven bone. Scale bars equal 2 cm (A), 1 mm (B), 100 µm (C–E).

The largest cortical fragment from Bonenburg (WMNM P88133), the thin sections produced from WMNM P-uncatalogued (probably derived from cranial material), and the numerous smaller unidentified cortical fragments (for which no precise anatomical placement is possible) share the same primary bone tissue and overall general features (Figs. 4, S5, S6). WMNM P88133 has a primary cortex rather similar to the previously discussed samples from Europe. However, observation of histology of this and most of the other Bonenburg samples is hampered by a nearly opaque outer diagenetic zone >2 mm wide (Figs. 4A, 4B). The remaining bone tissue is very well preserved. Macroscopically, the outer bone surface bears fine longitudinal striations (Figs. S4, S5A, S6A), of the same kind noted above for the Aust and Lilstock specimens (Fig. S3D).

As in the other specimens, vascularization is strictly longitudinal (Figs. 4A, 4B, S5B, S5C, S6B, S6C). Simple vascular canals and primary osteons are arranged in surface-parallel rows which may be enhanced by GM bordering and embracing the vascular canals (Figs. 4B, S6C). An external fundamental system does not appear to be present. The bone matrix, in which the vascular canals and primary osteons are set, is IFM, and together they form PIFT (Figs. 4C, S5C, S6C, S6D).

The GM show alternations of differently colored IFM but do not show an appreciable pattern in spacing, while they appear to show differences in fiber density (Figs. 4C, S6C, S6D). Under cross-polarized light, it is possible to observe clearly bright coarse fibers in the paler yellow areas, with a reduction of their presence corresponding with increased darkness in areas of darker brown color (Figs. 4B, 4C, S5C, S5D). The darkest GM seem to be made up by fewer intrinsic fibers (Fig. 4C).

Osteocyte lacunae are numerous in the IFM with mainly plump and irregularly shaped ones throughout tissue, while more flattened ones are scarcer and present only in centripetal lamellae of osteons (Fig. 4D). Osteocyte lacunar density and size is greater in the primary bone matrix compared to the lamellar bone of the osteons (Figs. 4D, S5D).

The largest fragment (WMNM P88133) is characterized by a continuous gradient in bone compactness from the inner more cancellous area to the outermost cortex (Fig. 4A), resulting from the advancement of the Haversian substitution and resorption fronts. A wide and diffuse Haversian substitution front is detectable toward the center of the section, evidenced by the interruption of the semicircular GM (Fig. 4B). Elsewhere in the section, secondary osteons develop preferentially within primary ones, conserving the primary arrangement of the osteon rows (Figs. 4B, 4C). Rarely, there are secondary and primary osteons showing an infill of centripetal layers of anisotropic woven bone (Fig. 4D) alongside osteons showing presence of intrinsic fibers similar to the ones of the surrounding IFM.

In the deep cortical area, there is a high amount of resorption cavities eroded into the compact bone which consist of primary bone only partially replaced by secondary remodeling. Resorption cavities are lined by lamellar bone resulting in an increasingly cancellous condition.

The longitudinal section (Fig. 4E) is dominated by simple longitudinal canals with a very limited degree of anastomosis. It is possible to observe diffuse strands of thin, dark fibers of variable length, distributed mainly longitudinally (Fig. 4E). Sometimes, the fibers intersect each other next to the edges of the vascular openings (Fig. 4E). The borders of secondary osteons are lined by bright lamellar bone, darker bone tissue, and by both types together in an alternating fashion.

### Histology and microanatomy of *Shastasaurus sikanniensis* holotype jaw bone samples

**Figure 5:**
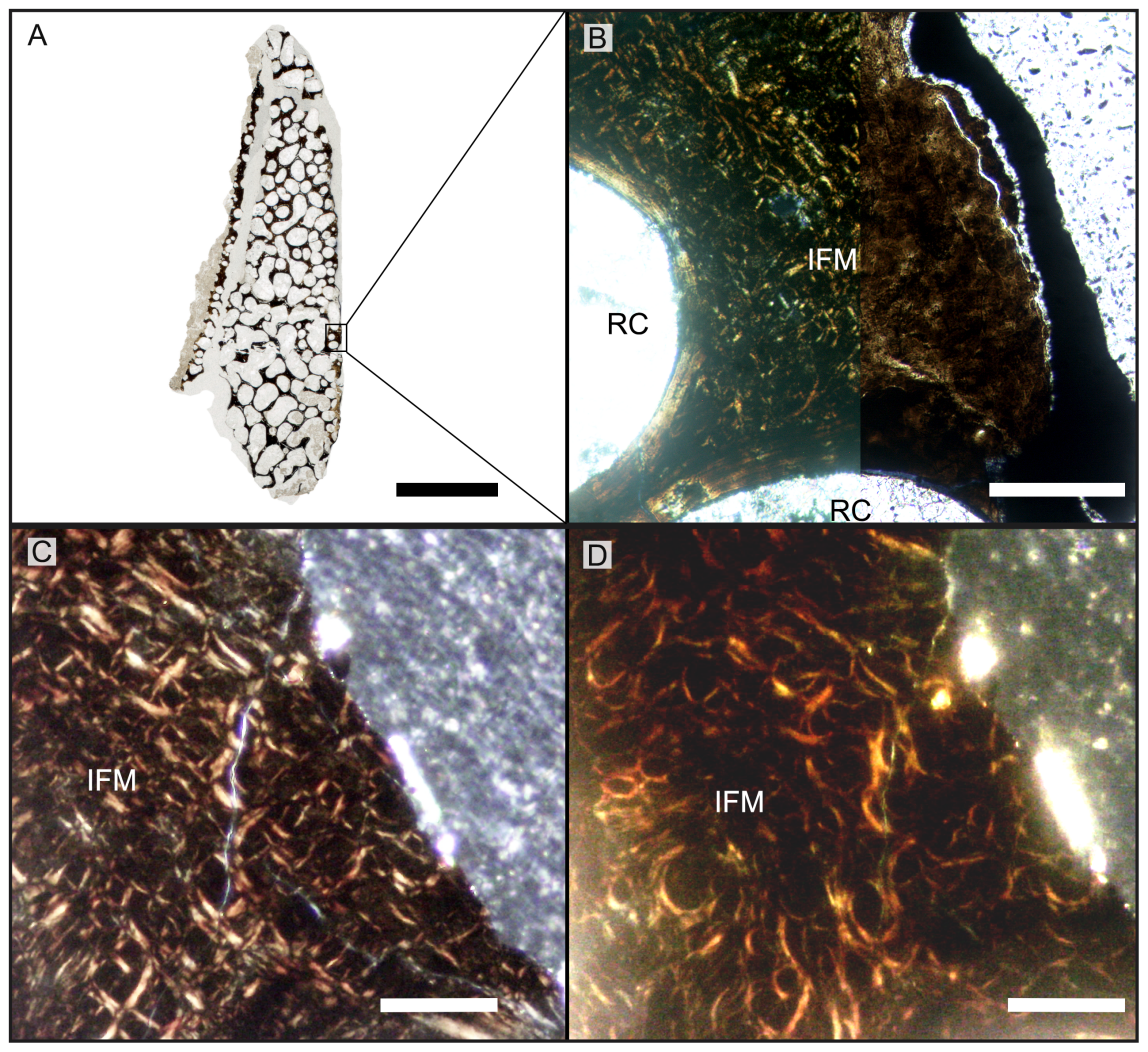
Histology of the sample from the splenial of the *Shastasaurus sikanniensis* type specimen RTMP-1994-378-0002 from the middle Norian of British Columbia, Canada. (A) Cross section of the splenial section (dorsal at top), the highly cancellous structure is evident, as well as the lack of a dense outer cortex, caused by taphonomic processes. (B) Close-up view of area indicated in (A). Primary cortex with IFM is preserved interstitially between secondary trabeculae. Left half of the image is in cross-polarized light, right half in normal light. Note the dark stain of the bone tissue in the normal-light image. Post-mortem, pre-burial erosion of the bone surface is indicated by the truncation of the bone structure and by the cover of opaque sediment. (C-D) Close-up showing IFM in cross-polarized light (C) and in circular polarized light (D). Note the helical arrangement of the fibers around a dark core. *Abbreviations*: IFM, intrinsic fiber matrix; RC, resorption cavity. Scale bars equal 5 mm (A), 100 µm (B), 50 µm (C, D).

Both of the jaw bone samples from the holotype of *S. sikanniensis* show poor histological preservation, which hides most of the discernable features in the areas where the bone tissue is most altered. This is particularly evident in the surangular. Poor preservation of birefringence is accompanied by a dark brown stain of the tissue, making it nearly opaque. However, with sufficiently bright illumination, the salient features, in particular the presence of IFM, can be discerned (Figs. 5B–5D). Both the surangular and splenial histology are characterized by highly spongious secondary bone tissue (porosity ∼82% and ∼60%, respectively), dark brown in color under the crossed polarizers (Fig. 5B). Towards the outer bone surface, which appears to be compromised by preparation (see below), there are interstitial areas of primary tissue characterized by distinctive IFM, with an outwards increase in frequency. Although no obvious dense cortical bone is present, a decrease in porosity is detectable toward the outer bone surface of both samples (respectively 64% and 43% porosity) with smaller longitudinal vascular cavities and higher compactness. The vascularization, consisting of large Haversian canals and resorption cavities, is strictly longitudinal (Figs. 5A, S3D, S7E, S7F). This is also seen with the naked eye on the outer bone surface which shows regular fine striations (Fig. S3E) as observed in the European specimens.

The secondary osteons visible in thin section have only few centripetal lamellae. On the outer bone surface, the presence of osteons half cut open indicates the removal of tissue due to taphonomic or diagenetic causes or harsh preparation (Figs. 5A, S3D). It is not possible to determine the presence of secondary osteons within primary ones in the trabecular bone. It is debatable, though, whether the absence of secondary osteons within primary ones is genuine or simply related to the lack of enough compact bone tissue in the sampled location of the jaw bones.

## Discussion

### Rejection of the “Dinosaur Hypothesis”

Although we do not question the ichthyosaurian status of the Lilstock and Autun specimens based on their morphology (Fischer et al., 2014; Lomax et al., 2018), the morphological information provided by the more fragmentary specimens (BRSMG-Cb-3869, BRSMG-Cb-3870, BRSMG-Cb-4063 from the UK and WMNM P88133 from Bonenburg, Germany) is insufficient for recognizing their systematic affinities (beyond excluding certain identifications, like as ichthyosaur or plesiosaur long bones). Thus, a comprehensive histological comparison was needed. Based on the histological evidence obtained from sampling bonafide (*i.e., S. sikanniensis*) and putative Late Triassic giant ichthyosaurs, we regard as relevant four histological features (Table 2), three of which had already been noted in the histological study of two Aust Cliff samples by Redelstorff, Sander & Galton (2014). IFM had not been reported by these authors, but was recognized by us in the same Aust Cliff thin sections.

All four features (Table 2) are present in the Lilstock and Autun ichthyosaur jaw specimens from the European Rhaetian. The most distinctive feature, IFM, is present in the jaw of the type specimen of the best-known giant ichthyosaur, *S. sikanniensis.* The uniqueness of IFM thus provides strong support for the “Giant Ichthyosaur Hypothesis”. Redelstorff, Sander & Galton (2014) concluded that the histology of the Aust Cliff bone segments did not resemble the histology of any dinosaur long bones known at the time. This statement still holds true, especially since the histology of virtually all dinosaur clades, and especially all large-bodied ones, is known by now. We thus can confidently reject the Dinosaur Hypothesis.

### Testing other possible affinities using histology

To test for the presence of a similar combination of features and to further test the “Giant Ichthyosaur Hypothesis”, we performed extensive histological comparisons, considering a “Non-dinosaur Hypothesis”, addressing known large or giant Late Triassic tetrapods, both terrestrial and aquatic. The results of this comparison are summarized in Table 2 and discussed in more detail below. We found that the unique combination of histological features of the Aust Cliff bone segments and German cortical fragments, combined with their large size (Fig. S3B), thick cortices, and shaft-like shape, rules out affinities with any other Late Triassic giant tetrapod, dinosaurian or non-dinosaurian, other than giant ichthyosaurs.

#### Archosauriformes

Among archosaurs, Crurotarsi presents Late Triassic forms with a generally S-shaped but sometimes straighter femur morphology, and it is possible that giant forms would have evolved straight propodial bones as seen in dicynodonts (Sulej & Niedźwiedzki, 2019), although convincing finds are lacking. The histology of large rauisuchians has been described in two genera, *Postosuchus* (4–5 m body length) and *Batrachotomus* (6 m). In *Postosuchus* from the Late Triassic of Texas, the femur shows a coexistence of lamellar-zonal tissue and a woven-parallel complex with sub-plexiform to laminar organization, while the outer cortex is lamellar-zonal (Ricqlés, Padian & Horner, 2003; Ricqlés, Buffrénil & Laurin, 2021). Therefore, the pattern and degree of vascularization and the abundance of lamellar-zonal tissue are not compatible with our observations.

The histology of *Batrachotomus* (Klein, Foth & Schoch, 2017) is also discussed here, despite its considerably greater geologic age (Ladininan, Middle Triassic), because of its presumed acquisition of gigantism through an increase in growth rate (Klein, Foth & Schoch, 2017) *Batrachotomus* thus exemplifies a hypothetical, fast-growing Rhaetian giant rauisuchian. The femur of *Batrachotomus* exhibits a highly vascularized woven-parallel complex that is more highly vascularized than that of *Postosuchus*, but the vascular organization is laminar to sub-plexiform, and secondary remodeling is rare, represented only by incipient secondary osteons (Klein, Foth & Schoch, 2017). These features are significantly different from the woven-parallel complex with longitudinal osteons and the strong secondary remodeling in the deep cortex we observed. Thus, we conclude that even a plausible giant, fast-growing rauisuchian must be excluded from consideration.

Although much smaller, aetosaurs show some superficial similarity in histologic features (Buffrénil, Quilhac & Cubo, 2021), but can easily be excluded. Aetosaur histology shows a general predominance of lamellar-zonal tissue or a laminar woven-parallel complex transitioning outward to poorly to non-vascularized lamellar-zonal tissue (Ricqlés, Padian & Horner, 2003; Buffrénil, Quilhac & Cubo, 2021), a less vascularization, and less well-organized secondary remodeling (Ricqlés, Padian & Horner, 2003; Buffrénil, Quilhac & Cubo, 2021).

Histology of the femora of large phytosaur specimens shows lamellar-zonal bone tissue (Ricqlés, Padian & Horner, 2003; Buffrénil, Quilhac & Cubo, 2021). In addition, a gradual decrease in vascularization toward the outer cortex, a poorly vascularized lamellar-zonal outer cortex, and scattered, unorganized secondary remodeling of the cortex have been reported (Ricqlés, Padian & Horner, 2003; Ricqlés, Buffrénil & Laurin, 2021). These features are marginally consistent with those reported in this work, but more specimens would have to be added to the comparison sample. However, IFM and secondary osteons within primary osteons have not been reported for any of the Crurotarsi considered, and for this and the other reasons listed above, we have excluded them from further consideration.

#### Triassic non-mammalian synapsids

Among Triassic non-mammalian synapsids, kannemeyeriiform dicynodonts are known to have reached large to giant sizes (3–4 m) in the Late Triassic (Benton, 2015; Sulej & Niedzwiedzki, 2019). Such animals had already been excluded by previous authors in the context of the “mystery bones” based on the morphology of the long bones (Redelstorff, Sander & Galton, 2014). The histology of the kannemeyeriiforms is somewhat closer to our samples in that they have a woven-parallel complex with longitudinal osteons often bordered by GM (Chinsamy & Rubidge, 1993; Green, Schweitzer & Lamm, 2010; Botha & Huttenlocker, 2021), but the vascularization of the large *Placerias* specimens appears to be less than that of the “mystery bones”. Moreover, the avascular or nearly avascular outer cortices of lamellar bone reported for both propodials and epipodials, together with the presence of scattered and rather chaotically arranged secondary osteons (Green, Schweitzer & Lamm, 2010; Botha & Huttenlocker, 2021), contrast with the outer cortical vascularization and characteristic secondary remodeling (including secondary osteons within primary osteons) observed in our specimens. Due to its large size, the largest known Kanemeyeriiform dicynodont, *Lisowicia bojani*, is more highly vascularized, but does not show the same Haversian organization in ordered periosteal rows as in our specimens (Sulej & Niedźwiedzki, 2019, fig. s14). Finally, the presence of an IFM-like primary matrix has not been reported for *Placerias*, *Kannemeyeria*, or *Lisowicia* (Chinsamy & Rubidge, 1993; Green, Schweitzer & Lamm, 2010; Sulej & Niedźwiedzki, 2019).

#### Sauropterygians

Sauropterygians were an important component of the Late Triassic faunas, but it would be difficult to identify representatives with bones of the size range of the specimens studied. Large rib specimens of *Nothosaurus* show a tissue rich in extrinsic fibers superficially resembling IFM and longitudinal vascular canals (Klein, Canoville & Houssaye, 2019, fig. 4k, n, o), but other *Nothosaurus* ribs show parallel-fibered bone tissue and radial vascularization (Klein, Canoville & Houssaye, 2019, fig. 4l), so the occurrence of an IFM-like matrix and longitudinal vascularization does not appear to be consistent within the genus. The histology of plesiosaurs, including the only Triassic one, has recently been extensively studied (Wintrich et al., 2017; Sander & Wintrich, 2021), but the complete lack of dermatocranial samples (Sander & Wintrich, 2021) for comparison prevents us from testing the hypothesis of a large unknown Triassic form. The histology of plesiosaur propodials, which are dominated by radial vascularization at mid-shaft (Wintrich et al., 2017; Sander & Wintrich, 2021), is certainly not consistent with the results of this study, but interestingly, secondary remodeling in plesiosaurs appears to follow pre-existing radial canals (Sander & Wintrich, 2021, p. 449), similar to what we described as template remodeling.

#### Temnospondyl amphibians

Recently, the idea that many temnospondyl clades may have persisted, even with large-bodied forms, until the very end of the Triassic has been proposed (Steyer & Damiani, 2005; Sander et al., 2016; Konietzko-Meier et al., 2018). Considering also the first attribution of the Aust Cliff bones to ‘Labyrinthodontia’ (Stutchbury, 1850), it seems appropriate to include temnospondyls in our comparison.

Late Triassic *Metoposaurus* mandibles (Gruntmeijer, Bodzioch & Konietzko-Meier, 2021) and long bones (Konietzko-Meier & Sander, 2013), as well as an indeterminate Late Triassic temnospondyl humerus (Konietzko-Meier et al., 2018), show the diffuse presence of an IFM-like bone matrix in the bone cortices, as well as a generally longitudinal orientation of the vascular canals. The poor primary vascularization and the rather disorganized and scattered secondary remodeling (Konietzko-Meier & Sander, 2013; Konietzko-Meier et al., 2018; Gruntmeijer, Bodzioch & Konietzko-Meier, 2021) are different from our specimens, but Konietzko-Meier et al. (2018) report that “in temnospondyls, the remodeling process always follows the vascular pattern of the primary tissue, unlike in Amniota....”. Nevertheless, the differences in morphology and histology are too great between the material studied here and temnospondyls to support such an affinity.

### IFM and PIFT and possible analogs

Although PIFT has not been explicitly described in the literature, it is not uncommon to see published micrographs seemingly showing this type of bone tissue or similar ones. A brief, but probably incomplete, list of examples includes a wide variety of amniotes: the rib sample of a large *Nothosaurus* specimen (Klein, Canoville & Houssaye, 2019, fig. 4n, o) (Figs. S8A, S8B), the femur of *Simosaurus* (Klein & Griebeler, 2016, fig. 5) (Figs. S8H, S8I), various bones of the thalattosaur *Askeptosaurus* (Klein et al., 2023) (Figs. S8D, S8E), the rib of the thalattosuchian crocodylomorph *Metriorhynchus* (Buffrénil, Quilhac & Cubo, 2021 fig. 10.2f), and the humerus of the ornithopod dinosaur *Telmatosaurus* (Buffrénil & Quilhac, 2021a, fig. 8.6a). Some of the more suggestive cases noted above are discussed in Article S2 and figured in Fig. S8.

### IFM, PIFT and ossified tendons

IFM remarkably resembles extrinsic fibers bundles seen in metaplastic bone tissue of osteoderms (Scheyer & Sander, 2004) and longitudinal fiber bundles of ossified tendons of various dinosaurs (Horner, Woodward & Bailleul, 2016; Surmik et al., 2023). However, we introduced the new terms IFM and PIFT in order to set this clearly periosteal matrix and tissue apart from metaplastic tissues. Nevertheless the similarity of PIFT with metaplastic bone tissue would suggest a shared osteogenic process.

Aside from the similarity between IFM and longitudinal extrinsic fibers, it is possible to observe further similarities with ossified tendons. The longitudinal strands of unmineralized fibers are in a herringbone pattern in both cases (compare Figs. 1E, 4E, S5D and, *e.g.*, Horner, Woodward & Bailleul, 2016 fig. 2g, Surmik et al., 2023 fig. 2f), and there are numerous irregular, sometimes elongate cell lacunae somewhat resembling fibrocytes in shape (Horner, Woodward & Bailleul, 2016). Finally, it is possible to see the presence of occasional centripetal coarse fibrous bone in the Haversian canals of *Meleagris gallopavo* tendon, figured by Adams & Organ (2005, figs. 2c, d).

The GM in our specimens find close similarity with the structures reported by Horner, Woodward & Bailleul (2016) as regions of varying primary orientation and density of the fibers (Horner, Woodward & Bailleul, 2016 fig. 2d-f). The hypothesis proposed by Horner, Woodward & Bailleul (2016), that the variable color of similar structures is related to the density and orientation of fibers observed in longitudinal sections in ossified tendons, fits our observations (Figs. S6C, S6D) and explains the appearance of such marks. Contrary to what was reported by Horner, Woodward & Bailleul (2016) for ossified tendons, the GM are identifiable as classical cycles of periosteal apposition, given the clear primary origin of these structures in relation to the spatial distribution of periosteal vascular canals and nutrient foramina, and the presence of osteocyte lacunae.

In conclusion, our literature review suggests that IFM is a novel matrix type that has not been reported before in the osteohistological literature. This leads to the question if a bone tissue formed of IFM may be viewed as an apomorphy of a clade of giant ichthyosaurs. This hypothesis would have to be tested by phylogenetic analysis incorporating histological characters, which may well find IFM as a non-unique synapomorphy, resulting from parallel osteogenetic processes. Alternatively, IFM could be mapped on an amniote cladogram and may show up as a synapomorphy.

### Template remodeling, osteons within osteons, and unmineralized fibrous matrices

The phenomenon of template remodeling, resulting from the formation of secondary osteons within primary ones, and secondary osteons within secondary osteons (*i.e.,* concentric osteons) appears to be a distinctive shared feature of the histology of the European samples investigated here. The unifying feature of all of these types of secondary remodeling is the reuse by the basic structural unit (bone remodeling unit) of preexisting vascular pathways, be they primary or themselves the result of previous remodeling activity. This reuse of existing pathways is unusual for secondary osteons which in dinosaurs, mammals, and most other amniotes show little regard for the primary histology (Mitchell, 2017). The diameter of secondary osteons in amniotes in general (Mitchell, 2017) is usually substantially greater than that of the primary osteons, resulting in the obliteration of primary features of histology (*e.g.*, Rogers & Kulik, 2018, fig. 4c and several others) and not in templating. We here emphasize the unusual nature of the secondary osteons within primary ones as a pattern we observe. Whereas this pattern may be a special feature of the specimens investigated here, it also could result from the strictly longitudinal orientation of the primary osteons or from the underlying propensity of reuse of vascular pathways. Although we are currently uncertain which of these mechanisms is at work, this does not diminish the systematic value of the secondary osteons within the primary ones.

As already noted, bone remodeling involving pre-existing primary or secondary osteons has been reported in various aquatic tetrapod taxa, such as in the long bones of plesiosaurs (Sander & Wintrich, 2021) and in temnospondyls (Konietzko-Meier et al., 2018). Klein et al. (2015) describe “secondarily widened primary osteons” (Klein et al., 2015 figs. 7, 8, s5) in various placodonts (Sauropterygia). With this term, they refer to the normal transformation process of compact bone to spongy bone. Specifically, these authors note that the resorption activity leading to cancellous bone, *i.e.,* the resorption front of Mitchell & Sander (2014), originates from pre-existing vascular canals. In this way, there is a similarity to secondary osteons within primary osteons. The difference, however, is that in our material the secondary osteons within the primary ones do not lead to cancellous bone, but the tissue remains compact. The transformation to cancellous bone occurs deeper in the cortex. An interesting report is that by Surmik et al. (2023) of what appear to be secondary osteons within primary osteons in ossified tendons of ornithischian dinosaurs (Surmik et al., 2023 fig. 2d). Although confirmation of the presence of this feature would require direct observation of the Surmik et al. (2023) sections, this potential occurrence may be informative on the underlying mechanism of vascular architecture reuse.

The occurrence of a common unusual feature in bone tissue formed by different processes (*e.g.*, periosteal apposition in mandibles and long bones *vs.* metaplastic ossification of ossified tendons and osteoderms) suggests a common constraint as explanation. In the process of bone resorption, osteoclasts are unable to act on the mineralized bone matrix until the organic protective layer of bone lining cells is removed by cambial cells (Zylberberg, 2021). It has also been hypothesized that sites characterized by non-mineralized structures are less attractive or accessible to osteoclasts (Aaron, 1980; Aaron, 2012; Jones, Boyde & Ali, 1984). The widespread presence of non-mineralized fibers in a bone tissue may significantly inhibit the progression of the basic structural units. The absence of unmineralized fibers in the osteonal bone matrix may thus induce primary osteons to serve as preferential “highways” for osteoclast activity, especially during the initial resorptive phase (*i.e.,* the resorption front), thus explaining the occurrence of abundant secondary osteons within primary osteons and template remodeling.

Alternatively, a difference between the regulatory signals emanating from osteocytes in the outer cortical matrix and those in the osteonal bone matrix may be the primary driver of osteoclast regulation and attraction. Osteocyte regulatory activity is known to be influenced by mechanical loading during development and appears to vary with lacunar shape (van Oers, Wang & Bacabac, 2015). Therefore, it is plausible that the numerous and highly heterogeneous lacunar spaces observed in the matrix may have played a critical additional role.

### Implications of PIFT for growth rate, gigantism, and feeding behavior

Several of the features we described are commonly associated with fast growth rates, the most common being a histology dominated by a woven-parallel complex (most commonly fibrolamellar bone), a high degree of vascularization, a high rate of remodeling with multiple generations of osteons, and a high number of osteocyte lacunae, both irregular and spindle-shaped (Buffrénil & Quilhac, 2021b). The presence of numerous open canals in the outer cortex and a well-vascularized outer periosteal surface indicates for all bones sampled that the animals were actively growing at the time of death. The presence of unmineralized fibers in the cortex could be related to rapid mineralization of the osteoid layer laid down by the periosteum as well as to the presence of fibrocytes (Buffrénil & Quilhac, 2021a). The latter cell type would be rather unusual in the formation of periosteal bone, however. The occasional presence of woven bone as infill of osteons may be another feature indicating rapid bone deposition.

Only limited hypotheses regarding developmental stages are possible, due to the absence of enough data to constraint precise taxonomic identity and the homologous location of the thin sections, except possibly for BRSMG-Cg-2488 R-101 and BRSMG-Cb-3869 (see Materials and Methods). In BRSMG 4063, the extension of dense Haversian tissue up to the outer cortex (Fig. 2D) may indicate a mature individual (Buffrénil & Quilhac, 2021b). It is equally likely, though, that the sample could originate from a region subject to high mechanical stress or mineral recycling (Buffrénil & Quilhac, 2021b). The size and number of the cortical vascular canals of BRSMG-Cg-2488 R-101 could indicate a younger, more actively growing individual compared to BRSMG-Cb-3869 (Buffrénil, Quilhac & Cubo, 2021). Before more samples and specimens become available to better understand the biology of these animals, we refrain from further speculations.

The similarity between the bone matrices of giant ichthyosaur mandibles, ossified tendons, and osteoderms invites speculations on the biomechanical properties of the former (as already done by Horner, Woodward & Bailleul (2016) with the hadrosaur nasal). For example, the largest bone segment from Aust Cliff has been suggested to belong to an animal in the size range of modern blue whales (Lomax et al., 2018). Although the feeding strategy of these giant ichthyosaurs remains unknown, it is reasonable to assume that their large jaws were adapted to withstand significant stress associated with hunting and feeding underwater, similar to the feeding behavior of blue whales, which actively process thousands of liters of seawater in one gulp (Goldbogen et al., 2011). Given the high tensile strength of mineralized ossified tendons, it is possible that these large ichthyosaur jaws were selected to withstand similar stresses, either during simple opening, as in baleen whales, or during potential ramming behavior, as observed in odontocetes such as killer whales. At the same time, the high amount of unmineralized fibers in the longitudinal axis of the mandible would have provided some flexibility in different bending planes (Horner, Woodward & Bailleul, 2016). The high rate of remodeling, typically associated with bones subjected to loading, is another factor supporting this hypothesis. It is possible that the presence of specialized soft tissues, such as muscle and connective tissue, likely played an important role in the development of this peculiar histology (Organ & Adams, 2005; Klein, Christian & Sander, 2012; Horner, Woodward & Bailleul, 2016). The occurrence of specializations for buccal processing of large amounts of water (relative to body size) is not isolated within Ichthyosauromorpha (Fang et al., 2023) and is expected in the evolutionary context of achieving giant sizes in marine environments (Sander et al., 2021).

## Conclusions

Paleohistology can be a powerful tool for determining the taxonomic affinity of fragmentary bone specimens, as has been demonstrated in dinosaur studies previously (*e.g.*, Garilli et al., 2009; Hurum et al., 2006). However, paleohistology can also be used to show that dinosaur-sized fragmentary bones do not belong to dinosaurs at all. Our study does just that, ruling out Sauropodomorpha and Stegosauria as possible sources of the mysterious large bone segments and fragments found in the European Rhaetian, thus rejecting the Dinosaur Hypothesis and instead supporting the Giant Ichthyosaur Hypothesis laid out by Lomax et al. (2018).

There are four distinctive histologic features common to the very large indeterminate bone segments and cortical fragments from the European Rhaetian: (1) IFM, (2) strictly longitudinal vascular architecture in the primary cortex, (3) closely spaced skeletal growth marks structuring primary osteons and vascular canals, and (4) abundance of secondary osteons within primary osteons. While IFM as a type of woven-fibered matrix and secondary osteons within primary osteons have rarely been observed in amniotes, the combination of all four features is unique to the material sampled here, and even small fragments of bone cortex, *e.g.*, from Bonenburg, Germany, are diagnostic. The same four histologic features are present in giant ichthyosaur jaw bones from the Rhaetian of the UK (Lilstock) and France (Autun). Two of the features, the unique IFM and the strictly longitudinal vascular architecture, is also seen in the jaw bones of the giant ichthyosaur *Shastasaurus sikanniensis* from the middle Norian of Canada. The four features in combination are absent in dinosaur histological samples, and two, IFM and secondary osteons within primary osteons (as a pervasive pattern), are not known from dinosaur histology. Similarly, we reject any affinities with hypothetical giant Crurotarsi, Kannemeyeriiformes, and Plesiosauria. We note some similarities with other secondarily aquatic tetrapods (Temnospondyli, thalattosaurs and possibly large nothosaurs), but these groups are also rejected due to significant size and morphological differences.

The histology reported here thus can be used to reliably identify cortical bone segments as belonging to giant ichthyosaurs, overcoming the problem of scarce morphological evidence. We conclude that the large bone segments from Aust Cliff are indeed fragments of giant ichthyosaur jaws, as are the cortical fragments from Bonenburg. WMNM P88133 and WMNM P-uncatalogued indicate animals comparable in size to the British and French mandibular fragments, suggesting the potential for similar discoveries of very large-bodied ichthyosaurs in the Exter Formation of northern Germany.

The common occurrence of a unique bone matrix type, IFM, in several giant Late Triassic ichthyosaurs indicates a shared ossification strategy in their lower jaws. IFM appears to be associated with closely spaced growth marks that show rhythmic changes in bone formation and template remodeling produced by the reuse of existing vascular architecture by the basic structural unit during remodeling. These features may be apomorphic for a clade of giant ichthyosaurs and/or related to specific biomechanical properties of their mandibles. More comparative histological samples of ichthyosaurs and more complete specimens are needed to confirm these hypotheses.

Finally, our study shows that there still are novel bone matrix and bone tissue types to be discovered that are restricted to a specific extinct clade. IFM and PIFT are apparently extinct, and future work must address the evolutionary, phylogenetic, and developmental dynamics associated with the nature of IFM and its possible unrecognized presence in modern animals and the fossil record, and the reasons for its strong resemblance to the products of metaplastic ossification of extrinsic fibers, despite IFM being composed of intrinsic fibers in the periosteal territory.

## Acknowledgements

The authors are deeply indebted to Deborah Hutchinson and Roger Vaughan (BRSMG), Valentin Fischer (University of Liège, Belgium), Brandon Strelitzky and Don Brinkman (Royal Tyrrell Museum of Paleontology, Drumheller, Alberta, Canada), and Achim Schwerman (Westphalian Museum of Natural History, Münster, Germany) for access, permission to sample, and assistance with photographs and information about the specimens in their care. Olaf Dülfer and Pia Schucht (University of Bonn, Germany) are thanked for assistance and preparation of the thin sections. René-Paul Eustache (Combon, France) helped us to modify the Leica polarizing microscope for circular polarization. We thank Dorota Konietzko-Meier and Sudipta Kalita (University of Bonn, Germany), Peter Galton (University of Bridgeport, Connecticut, USA), Dean Lomax (University of Manchester, UK), and Paul De la Salle (The Etches Collection, Wareham, UK) for information and discussions. Many thanks to Andrzej Wolniewicz (Polish Academy of Sciences, Warsaw, Poland) for pointing out the von Huene study (1912). Finally, we would like to thank the editor, Mark Young, and the three reviewers (Megan Withney, Christopher Griffin, and an anonymous reviewer) for their work and useful comments that greatly improved this manuscript.

## Supplemental Information

10.7717/peerj.17060/supp-1 Supplemental Information 1 Map of western and central European localities source of studied material Purple stars indicate the source of Rhaetian specimens of this study. Inset shows paleogeographic reconstruction of Europe and the Western Tethys in the Rhaetian (modified from (Schobben et al., 2019); CC BY-NC 3.0 DEED). The red and green marks show the approximate position of investigated fossil localities in the shallow marine environments. *Abbreviations*: CEB, Central European Basin; RM, Rhenish Massif.

10.7717/peerj.17060/supp-2 Supplemental Information 2 British and French specimens and their derived thin sections Photos of the sampled specimens (A) and scans of the resulting thin section B, all to the same scale. White circles indicate coring location. White arrows point at foramina identified as part of *fossa surangularis* by (Lomax et al., 2018). Thin sections were cut in a transverse plane of the bone, representing parts of the bone cross section. Scale bars represent 10 cm (A), 2 mm (B).

10.7717/peerj.17060/supp-3 Supplemental Information 3 Overview of described material assigned to giant ichthyosaurs Ventral view of the skull of the *S.sikanniensis* holotype RTMP-1994-378-0002 from the middle Norian of British Columbia, Canada, with sampling location. Red circles indicate sample location on the surangular (light blue), and splenial (yellow). Dotted line indicates unsure border between bones. (B) Kuleuven PLV-1964 (top) from Autun, France, and BRSMG-Cg-2488 (bottom, from Lomax et al., 2018, CC0), from Lilstok, UK. The segment sampled from the Lilstock specimen (BRSMG-Cg-2488-R101) is the distalmost one. (C) The 1.4 meter long specimen studied and described by Huene (1912) in medial (top) and lateral view (bottom) and cross section (side). Note the second sectioned fragment (light grey) possibly representing BRSMG Cb 3870. Modified after (Huene, 1912). (D) Detail of the surface of BRSMG-Cg-2488 showing longitudinal ridges and striations caused by the strictly longitudinal vascularization on the subperiosteal interface. Tooth marks are also visible, interrupting the continuity of the longitudinal ridges and striations (from Lomax et al., 2018), CC0). (E) Detail of the sampling area of RTMP-1994-378-0002 splenial. Note the ridges and striations identical to the ones in (D), and the higher worn state of the surface. The cross section allows to observe the continuity of the longitudinal vascularization with said striations. *Abbreviations*: An, angular; D, dentary; Sp, splenial; Sur, surangular. Scale bars represent 50 cm (A), 20 cm (B).

10.7717/peerj.17060/supp-4 Supplemental Information 4 WMNM P88133, the largest cortex fragment from the late middle Rhaetian locality of Bonenburg, Westphalia, Germany (A) Outer bone surface (top), side view (middle) and internal view (bottom). The internal surface shows strong signs of erosion that must have removed less compact bone tissue. Dotted line in white the reconstructed area indicates the plane of section. (B) Scan of the cross section (left) and longitudinal section (right). The outer bone surface is on the right, the internal surface is on the left. White dotted line indicates the plane of the longitudinal section. Scale bar represents five cm (A) and one cm (B).

10.7717/peerj.17060/supp-5 Supplemental Information 5 Example of a typical small fragment from Bonenburg (WMNM P88130 to P88144), and of their histology (A) External morphology from different views (from left to right: external, cross section, and internal). (B) Cross section in normal light, note the strictly longitudinal vascularization. (C) Cross section at higher magnification, IFM, secondary osteons within primary osteons and GM (left side in cross-polarized light, right side in normal light). (D) Longitudinal section, strands of unmineralized fibers running longitudinally in a herringbone pattern seen in normal light. *Abbreviations*: IFM, intrinsic fiber matrix. Scale bars represent: 5 mm (A); 1 mm (B); 100 µm (C, D).

10.7717/peerj.17060/supp-6 Supplemental Information 6 WMNM P-uncatalogued, another large cortical fragment from the bone bed of Bonenburg similar to WMNM P88133 (A) Top view of the outer surface, yellow line indicates location of section. (B) Composite image showing the general histology of the section, comparable to WMNM P88133 and to the more complete French and British material. (C) Close up in cross polarized light showing the border (white dotted line) between template deep cortex (top) and regular deep cortex (bottom). (D) Detail in cross polarized light, showing the presence of IFM and the difference in thickness of bright and darker GM in the lower half of image. White arrows indicate growth marks. *Abbreviations:* IFM, intrinsic fiber matrix. Scale bars represent: 5 cm (A); 1 mm (B, C); 100 µm (D).

10.7717/peerj.17060/supp-7 Supplemental Information 7 Binary micrographs produced from scans of thin sections for porosity evaluation (A) BRSMG-Cb-3869. (B) BRSMG-Cb-3870. (C) BRSMG-Cg-2488 R-101. (D) BRSMG-Cb-4063. (E) KULeuven PLV-1964. (F) Surangular of RTMP-1994-378-0002. (G) splenial of RTMP-1994-378-0002.

10.7717/peerj.17060/supp-8 Supplemental Information 8 Comparison of different coarse fibered bone tissues (A–B) *Nothosaurus* rib SMNS 80266 (reproduced after (Klein, Canoville & Houssaye, 2019). (C) UPFB in the humerus of the mosasaurus *Clidastes* sp., UCMP 34536 (reproduced after (Houssaye et al., 2013). (D) Cross polarized view of the CPFB in the femur of *Askeptosaurus italicus* PIMUZ 4839. (E) Detail in circular polarized light of the coarse fibers in PIMUZ 4839. (F) BRSMGCg-2488 showing PIFT in cross-polarized light. (G) cross-polarized light view of *Homalocephale calathocercos* MPC-D 100/1201 ossified tendon (reproduced after Surmik et al., 2023). (H–I) *Simosaurus* SMNS 91983 femur showing CPFB in the endosteal domain (reproduced from Klein & Griebeler, 2016 Copyright ©2016 ElsevierMasson SAS. All rights reserved.). (J) Circular polarized light views of *Diplodocus* SMA HQ2 cervical rib ossified tendon showing an outer surface of metaplastic tissue and large secondary osteons (see also Klein, Christian & Sander, 2012). (K) Circular polarized view of *Metoposaurus krasiejowensis* (UOPB 01145) lower jaw, showing coarse fibers on the left (see also Gruntmeijer, Bodzioch & Konietzko-Meier, 2021). (L) Cross (left) and circular (right) polarized light view of the humerus of a cyclotosaurian temnospondyl (WMNM P 64371) from Bonenburg (see also Konietzko-Meier et al., 2018). All scale bars equal 200 µm.

10.7717/peerj.17060/supp-9 Supplemental Information 9 Description of the specimens and areas of sampling

10.7717/peerj.17060/supp-10 Supplemental Information 10 Comparison of coarse fibered specimens in the litterature

## Additional Information and Declarations

### Data Availability

The following information was supplied regarding data availability: - BRSMG: Bristol Museum and Art Gallery. Queens Rd, Clifton, Bristol BS8 1RL, England. - IGPB: Histological collection, Institute for Geology and Paleontology Bonn, division Paleontology. Meckenheimer Allee 176, 53115 Bonn, Germany. - RTMP: Royal Tyrrell Museum. 1500 N Dinosaur Trail, Drumheller, AB T0J 0Y0, Canada *S. sikanniensis* holotype: Acc. Number RTMP-1994-378-0002; Specimen repository: RTMP; Thin section surangular (1) repository: IGPB; Thin section splenial (1) repository: IGPB. Lilstock putative ichthyosaur surangular; Acc. Number: BRSMG-Cg-2488 R-101; Specimen repository: BRSMG; Thin sections (2) repository: BRSMG. Aust Cliff Bone fragment; Acc. Number: BRSMG-Cb-3869; Specimen repository: BRSMG; Thin section (1): BRSMG. Aust Cliff Bone fragment; Acc. Number: BRSMG-Cb-3870; Specimen repository: BRSMG; Thin section (1): BRSMG. Aust Cliff Bone fragment; Acc. Number: BRSMG-Cb-4063; Specimen repository: BRSMG; Thin section (2): BRSMG. Autun putative surangular (2 fragments); Acc. Number: PLV-1964; Specimen repository: IGPB; Thin sections (2): IGPB. Small Bonenburg fragment; Acc. Number: WMNM P 88130; Specimen repository: IGPB; Thin sections (4): IGPB. Small Bonenburg fragment; Acc. Number: WMNM P 88131; Specimen repository: IGPB; Thin sections (5): IGPB. Small Bonenburg fragment; Acc. Number: WMNM P 88132; Specimen repository: IGPB; Thin sections (4): IGPB. Large Bonenburg fragment; Acc. Number: WMNM P 88133; Specimen repository: IGPB; Thin sections (4): IGPB. Small Bonenburg fragment; Acc. Number: WMNM P 88134; Specimen repository: IGPB; Thin sections (5): IGPB. Small Bonenburg fragment; Acc. Number: WMNM P 88135; Specimen repository: IGPB; Thin sections (4): IGPB. Small Bonenburg fragment; Acc. Number: WMNM P 88136; Specimen repository: IGPB; Thin sections (2): IGPB. Small Bonenburg fragment; Acc. Number: WMNM P 88137; Specimen repository: IGPB; Thin sections (2): IGPB. Small Bonenburg fragment; Acc. Number: WMNM P 88138; Specimen repository: IGPB; Thin sections (2): IGPB. Small Bonenburg fragment; Acc. Number: WMNM P 88139; Specimen repository: IGPB; Thin sections (2): IGPB. Small Bonenburg fragment; Acc. Number: WMNM P 88140; Specimen repository: IGPB; Thin sections (2): IGPB. Small Bonenburg fragment; Acc. Number: WMNM P 88141; Specimen repository: IGPB; Thin sections (2): IGPB. Small Bonenburg fragment; Acc. Number: WMNM P 88142; Specimen repository: IGPB; Thin sections (4): IGPB. Small Bonenburg fragment; Acc. Number: WMNM P 88143; Specimen repository: IGPB; Thin sections (2): IGPB. Small Bonenburg fragment; Acc. Number: WMNM P 88144; Specimen repository: IGPB; Thin sections (2): IGPB. Large Bonenburg fragment; Acc. Number: WMNM P Uncatalogued; Specimen repository: IGPB; Thin sections (2): IGPB.
