# Supplementary figures and images for: The dinosaurs that weren’t: osteohistology supports giant ichthyosaur affinity of enigmatic large bone segments from the European Rhaetian
Source: PeerJ. 2024 Apr 9;12:e17060. doi: 10.7717/peerj.17060 (PMC11011611; doi:10.7717/peerj.17060)

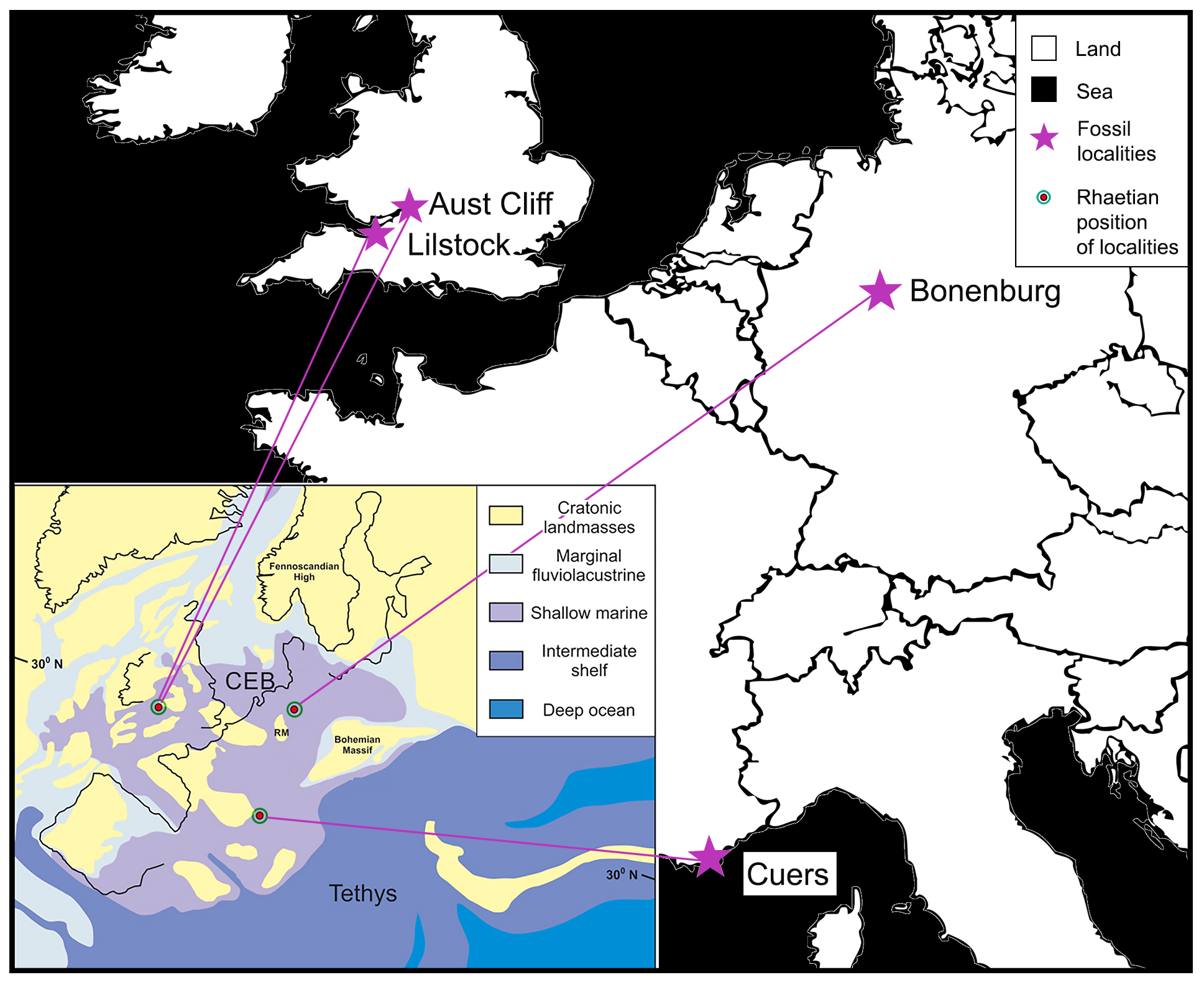

Supplement: Supplemental Information 1 — Purple stars indicate the source of Rhaetian specimens of this study. Inset shows paleogeographic reconstruction of Europe and the Western Tethys in the Rhaetian (modified from (Schobben et al., 2019); CC BY-NC 3.0 DEED). The red and green marks show the approximate position of investigated fossil localities in the shallow marine environments. Abbreviations: CEB, Central European Basin; RM, Rhenish Massif. [file peerj-12-17060-s001.png]

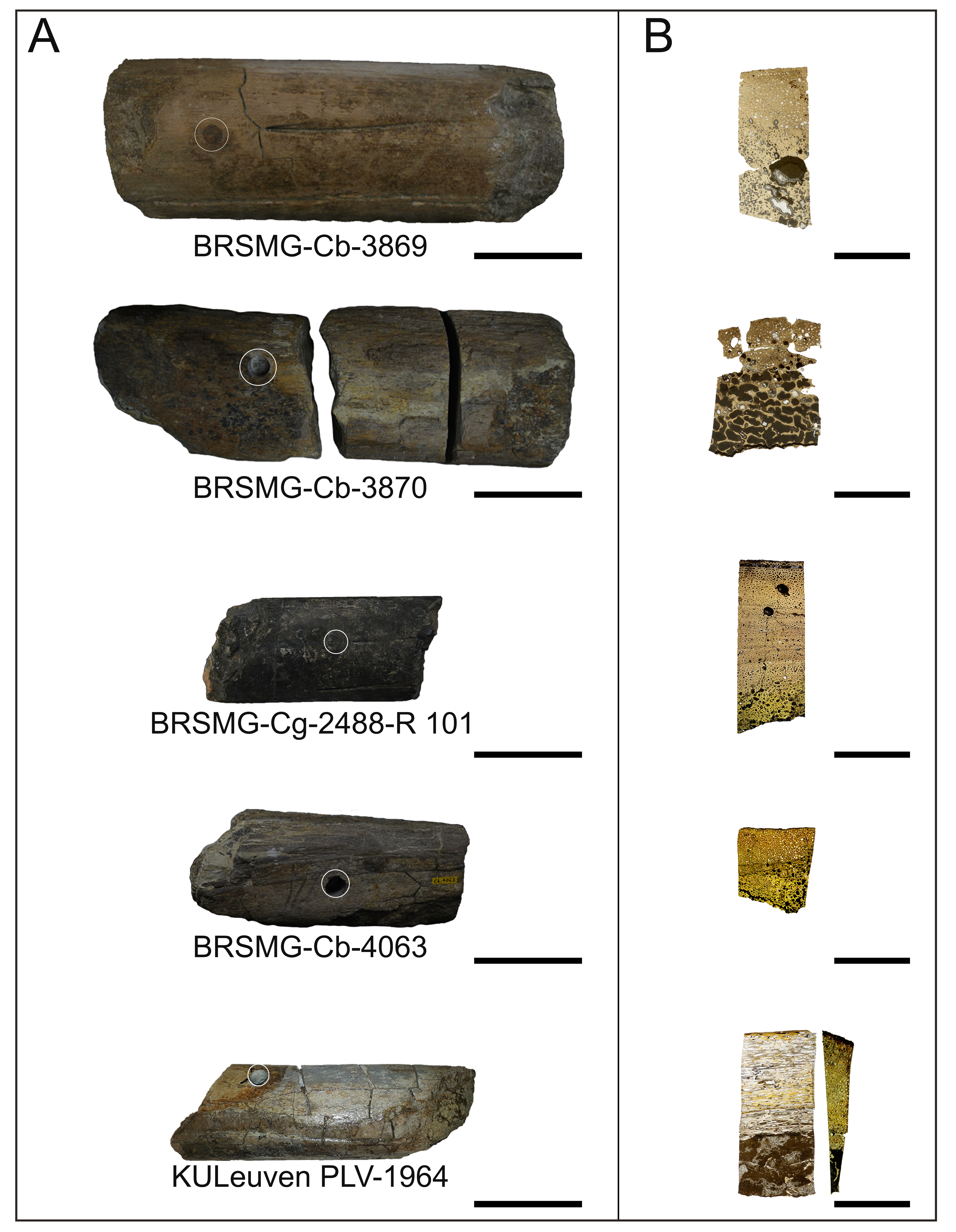

Supplement: Supplemental Information 2 — Photos of the sampled specimens (A) and scans of the resulting thin section B, all to the same scale. White circles indicate coring location. White arrows point at foramina identified as part of fossa surangularis by (Lomax et al., 2018). Thin sections were cut in a transverse plane of the bone, representing parts of the bone cross section. Scale bars represent 10 cm (A), 2 mm (B). [file peerj-12-17060-s002.png]

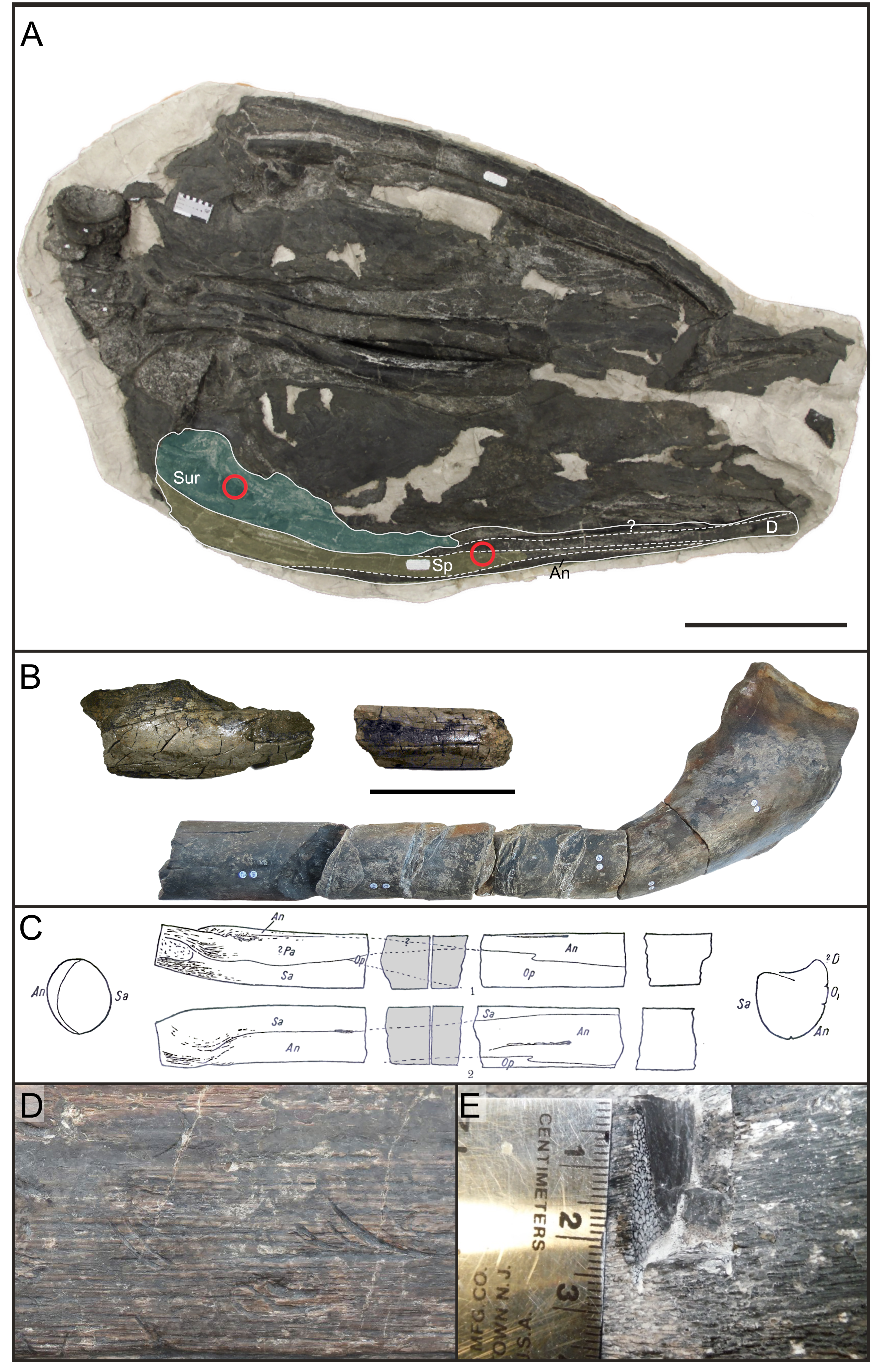

Supplement: Supplemental Information 3 — Ventral view of the skull of the S.sikanniensis holotype RTMP-1994-378-0002 from the middle Norian of British Columbia, Canada, with sampling location. Red circles indicate sample location on the surangular (light blue), and splenial (yellow). Dotted line indicates unsure border between bones. (B) Kuleuven PLV-1964 (top) from Autun, France, and BRSMG-Cg-2488 (bottom, from Lomax et al., 2018, CC0), from Lilstok, UK. The segment sampled from the Lilstock specimen (BRSMG-Cg-2488-R101) is the distalmost one. (C) The 1.4 meter long specimen studied and described by Huene (1912) in medial (top) and lateral view (bottom) and cross section (side). Note the second sectioned fragment (light grey) possibly representing BRSMG Cb 3870. Modified after (Huene, 1912). (D) Detail of the surface of BRSMG-Cg-2488 showing longitudinal ridges and striations caused by the strictly longitudinal vascularization on the subperiosteal interface. Tooth marks are also visible, interrupting the continuity of the longitudinal ridges and striations (from Lomax et al., 2018), CC0). (E) Detail of the sampling area of RTMP-1994-378-0002 splenial. Note the ridges and striations identical to the ones in (D), and the higher worn state of the surface. The cross section allows to observe the continuity of the longitudinal vascularization with said striations. Abbreviations: An, angular; D, dentary; Sp, splenial; Sur, surangular. Scale bars represent 50 cm (A), 20 cm (B). [file peerj-12-17060-s003.png]

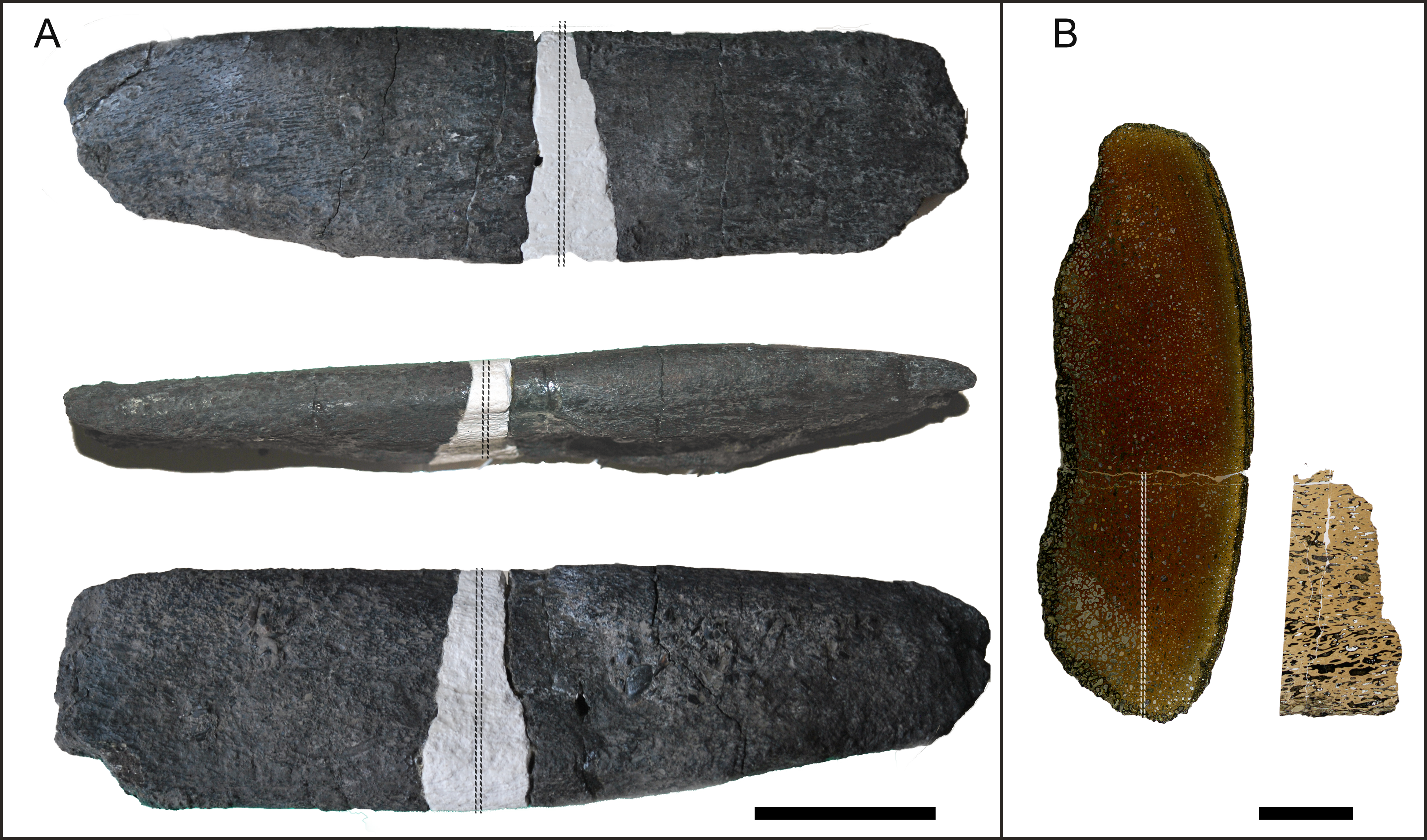

Supplement: Supplemental Information 4 — (A) Outer bone surface (top), side view (middle) and internal view (bottom). The internal surface shows strong signs of erosion that must have removed less compact bone tissue. Dotted line in white the reconstructed area indicates the plane of section. (B) Scan of the cross section (left) and longitudinal section (right). The outer bone surface is on the right, the internal surface is on the left. White dotted line indicates the plane of the longitudinal section. Scale bar represents five cm (A) and one cm (B). [file peerj-12-17060-s004.png]

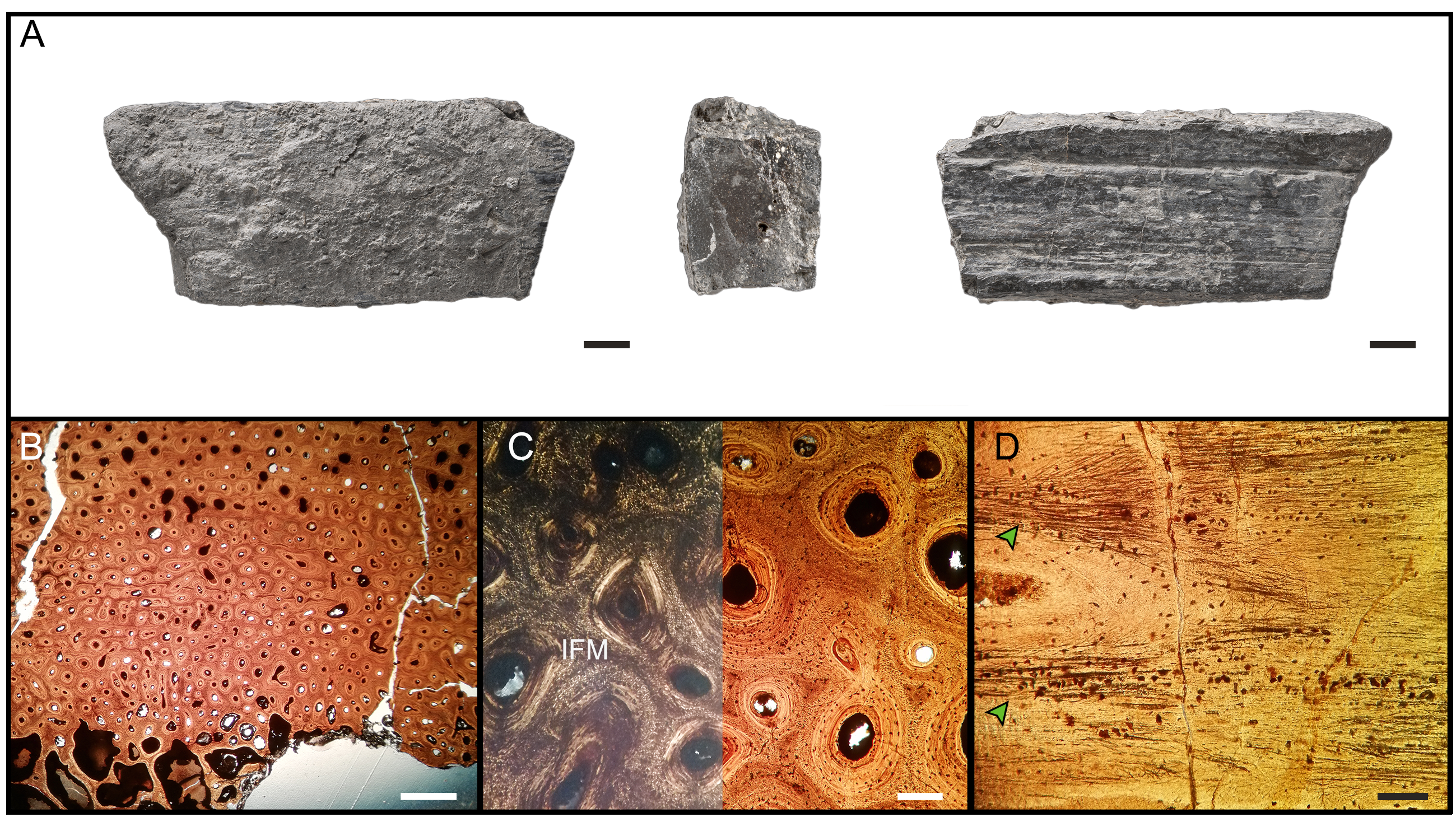

Supplement: Supplemental Information 5 — (A) External morphology from different views (from left to right: external, cross section, and internal). (B) Cross section in normal light, note the strictly longitudinal vascularization. (C) Cross section at higher magnification, IFM, secondary osteons within primary osteons and GM (left side in cross-polarized light, right side in normal light). (D) Longitudinal section, strands of unmineralized fibers running longitudinally in a herringbone pattern seen in normal light. Abbreviations: IFM, intrinsic fiber matrix. Scale bars represent: 5 mm (A); 1 mm (B); 100 µm (C, D). [file peerj-12-17060-s005.png]

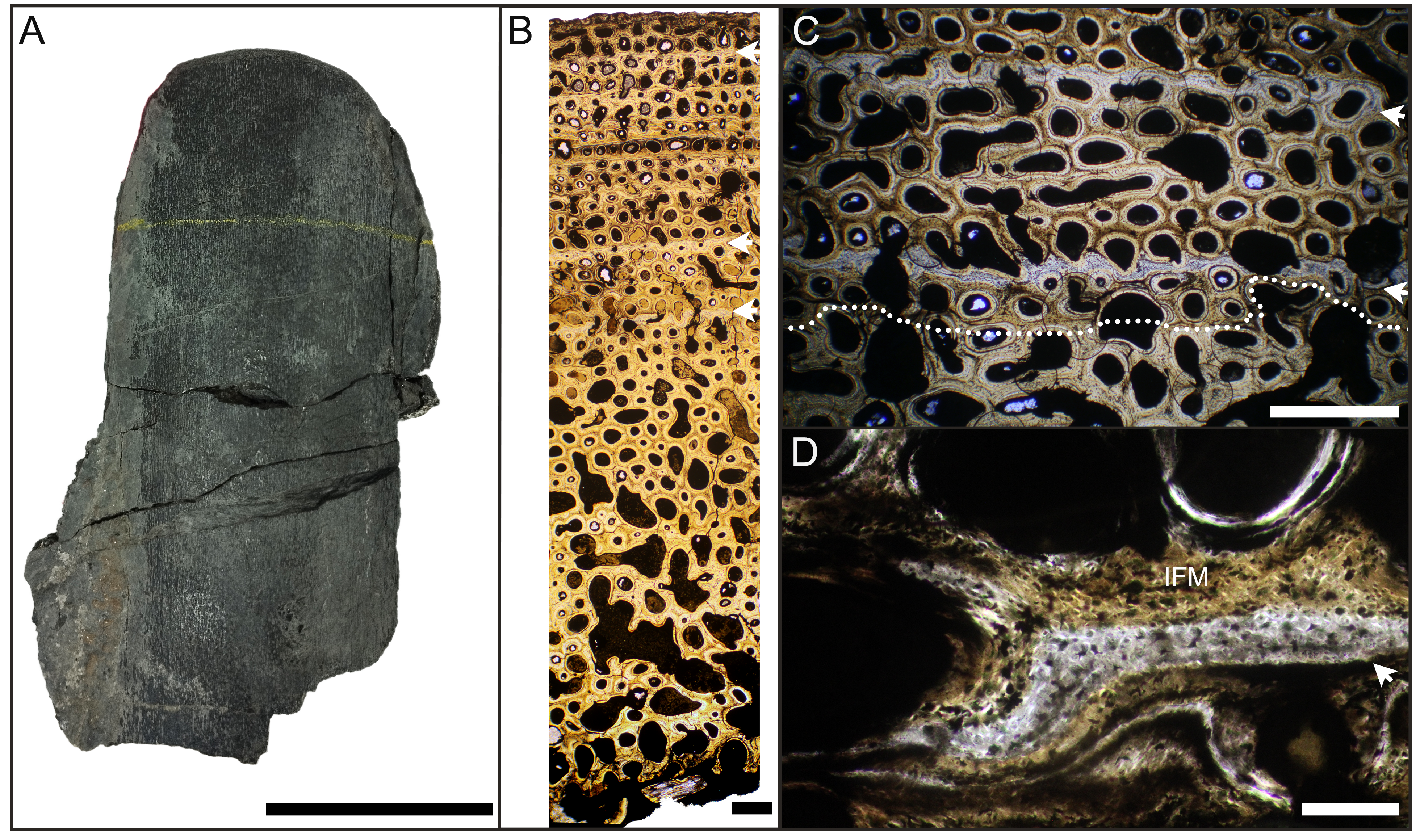

Supplement: Supplemental Information 6 — (A) Top view of the outer surface, yellow line indicates location of section. (B) Composite image showing the general histology of the section, comparable to WMNM P88133 and to the more complete French and British material. (C) Close up in cross polarized light showing the border (white dotted line) between template deep cortex (top) and regular deep cortex (bottom). (D) Detail in cross polarized light, showing the presence of IFM and the difference in thickness of bright and darker GM in the lower half of image. White arrows indicate growth marks. Abbreviations: IFM, intrinsic fiber matrix. Scale bars represent: 5 cm (A); 1 mm (B, C); 100 µm (D). [file peerj-12-17060-s006.png]

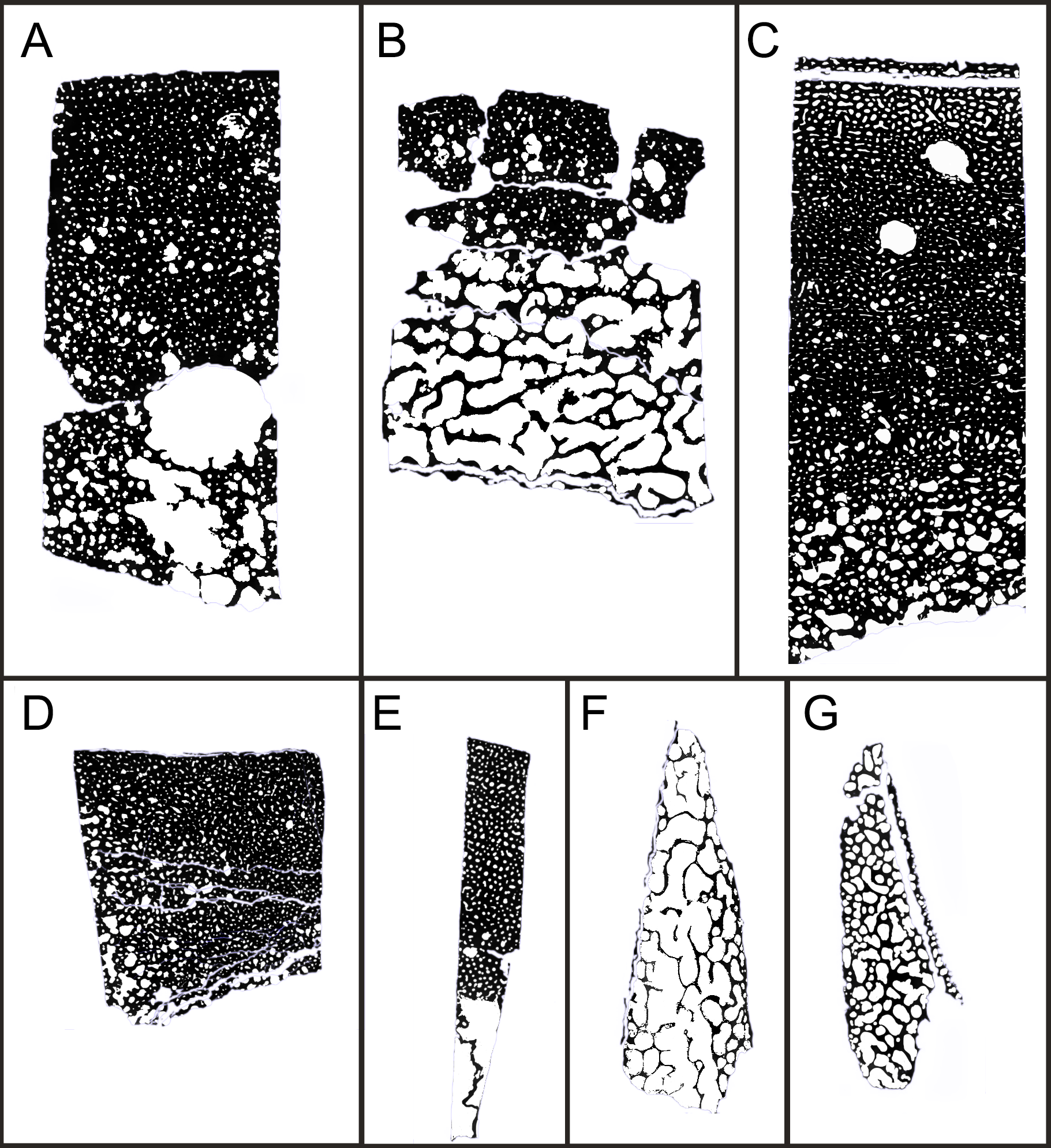

Supplement: Supplemental Information 7 — (A) BRSMG-Cb-3869. (B) BRSMG-Cb-3870. (C) BRSMG-Cg-2488 R-101. (D) BRSMG-Cb-4063. (E) KULeuven PLV-1964. (F) Surangular of RTMP-1994-378-0002. (G) splenial of RTMP-1994-378-0002. [file peerj-12-17060-s007.png]

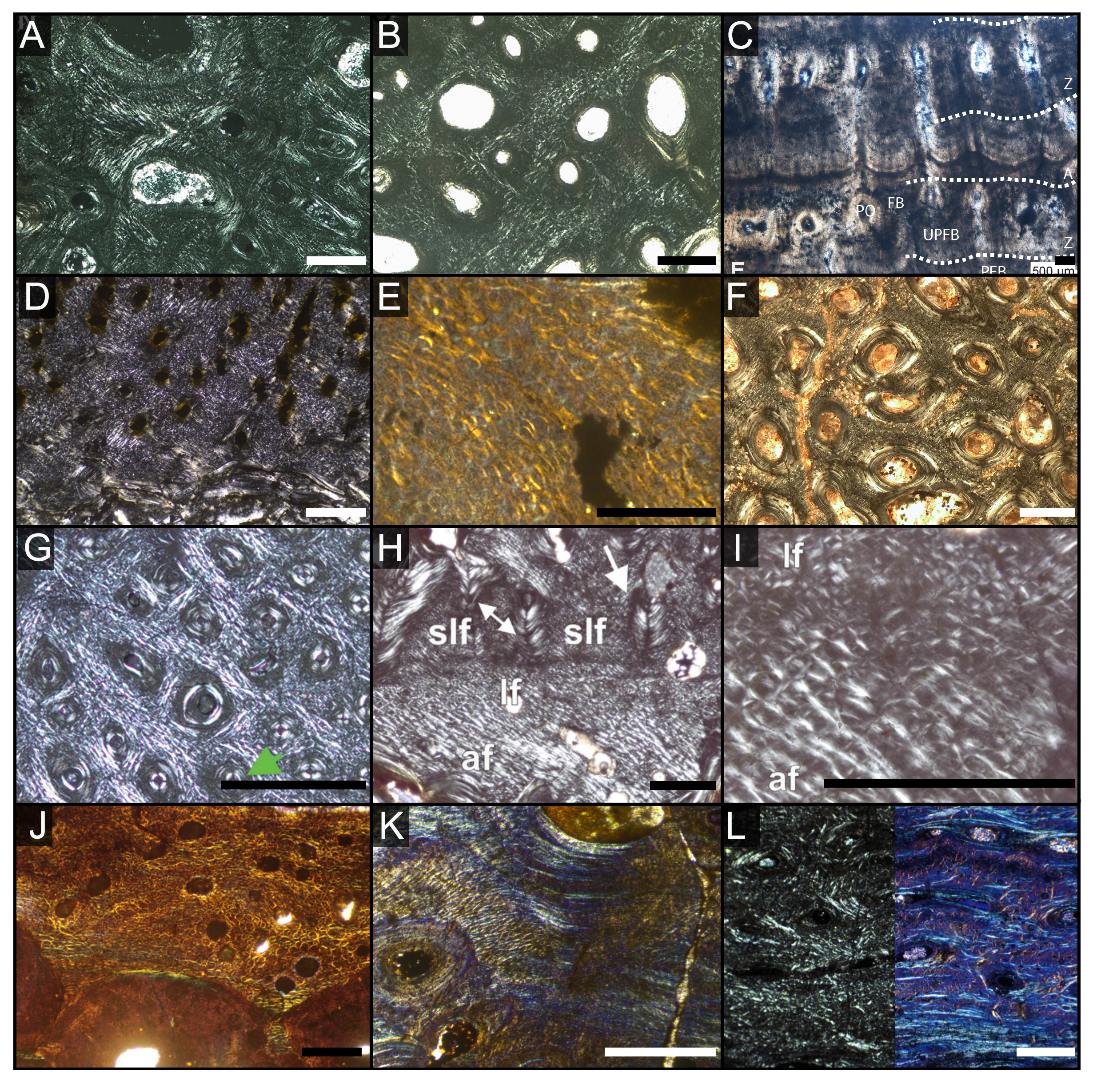

Supplement: Supplemental Information 8 — (A–B) Nothosaurus rib SMNS 80266 (reproduced after (Klein, Canoville & Houssaye, 2019). (C) UPFB in the humerus of the mosasaurus Clidastes sp., UCMP 34536 (reproduced after (Houssaye et al., 2013). (D) Cross polarized view of the CPFB in the femur of Askeptosaurus italicus PIMUZ 4839. (E) Detail in circular polarized light of the coarse fibers in PIMUZ 4839. (F) BRSMGCg-2488 showing PIFT in cross-polarized light. (G) cross-polarized light view of Homalocephale calathocercos MPC-D 100/1201 ossified tendon (reproduced after Surmik et al., 2023). (H–I) Simosaurus SMNS 91983 femur showing CPFB in the endosteal domain (reproduced from Klein & Griebeler, 2016 Copyright ©2016 ElsevierMasson SAS. All rights reserved.). (J) Circular polarized light views of Diplodocus SMA HQ2 cervical rib ossified tendon showing an outer surface of metaplastic tissue and large secondary osteons (see also Klein, Christian & Sander, 2012). (K) Circular polarized view of Metoposaurus krasiejowensis (UOPB 01145) lower jaw, showing coarse fibers on the left (see also Gruntmeijer, Bodzioch & Konietzko-Meier, 2021). (L) Cross (left) and circular (right) polarized light view of the humerus of a cyclotosaurian temnospondyl (WMNM P 64371) from Bonenburg (see also Konietzko-Meier et al., 2018). All scale bars equal 200 µm. [file peerj-12-17060-s008.png]
