# Supplementary material for: The dinosaurs that weren’t: osteohistology supports giant ichthyosaur affinity of enigmatic large bone segments from the European Rhaetian
Source: PeerJ. 2024 Apr 9;12:e17060. doi: 10.7717/peerj.17060 (PMC11011611; doi:10.7717/peerj.17060)
Supplement: Supplemental Information 9 [file peerj-12-17060-s009.docx]

Appendix S1. Description of studied specimens

# **Large bone segments from the Rhaetic Bonebeds of southwestern UK**

The material coming from the Rhaetian Westbury Formation of the Bristol Channel area (Aust Cliff and Lilstock). The litho- and chronostratigraphy of the Rhaetian and Hettangian deposits in the area is rather complicated. The Westbury Formation has the classical Rhaetic bonebed at is base but also has yield bones from near its top.  Westbury Formation is overlain by the Cotham Formation which is believed to contain the end-Triassic extinction events. Above the Cotham Formation, there are two more formations of Rhaetian age (White Lias and Watchet Formations). The age of all four formations is difficult to constrain beyond it being Rhaetian. Similarly, the location of the Triassic-Jurassic boundary has been controversial, but is generally believed to lie in the lower part of the Blue Lias Formation (see section in Lomax *et al.* 2018). The fossil material was subject to various previous studies, both morphological and histological ones (e.g. Galton 2005; Redelstorff *et al.* 2014; Lomax *et al.* 2018). Due to the lack of unequivocal diagnostic characters led to disparate conclusions regarding its nature (Fig. S1). The most recent hypothesis suggests the fragments to originate from lower jaws of giant ichthyosaurs (Lomax *et al.* 2018).

**BRSMG-Cb-3869 from Aust Cliff**

This specimen (Fig. S1A) is a single large (the largest by diameter of the sample) straight segment of bone recovered during the 19^th^ century from the Westbury Formation of Aust Cliff (Galton 2005). The discovery horizon within the Westbury Formation is unknown. The bone segment measures 12 cm in diameter. Both the original external cortex, as can be deduced by presence of vascular grooves and fossil boring traces (Lomax et al. 2018), and internal cancellous bone are preserved on the specimen. The section was produced from a drill core prior to this study and described by Redelstorff et al. (2014). Here we document the coring location (Fig. S1A) since no photo of the sampling location was published by Redelstorff et al. (2014). The core was extracted from the immediate proximity of an elongated furrow (Fig. S1A) which was mentioned by Lomax et al. (2018) as a possible homologue feature shared with BRSMG-Cg-2488 R-101.

**BRSMG-Cb-3870 from Aust Cliff**

This specimen consists of a straight segment of bone, recovered during the 19^th^ century from the Westbury Formation of Aust Cliff. The discovery horizon within the Westbury Formation is unknown. The segment is broken into two fragments, one of which was cut in half and polished in historical times (Galton 2005). Possibly the specimen was previously briefly described by von Huene (1912) (Fig. S2D). As for the previous specimen, the section was produced from a drill core prior to this study and described by Redelstorff *et al*. (2014). Here we document the coring location (Fig. S1A).

**BRSMG-Cb-4063 from Aust Cliff**

This specimen is a single straight bone segment found at Aust Cliff in the 19^th^ century and figured by Galton (2005). The discovery horizon within the Westbury Formation is unknown., The specimen, although being part of the Bristol bone segments collection and considered by Lomax *et al.* (2018) in the context of the ‘Giant Ichthyosaur’ hypothesis, was not subject to previous histological study by Redelstorrf *et al.* (2014); therefore it was sampled and studied to cover this gap. BRSMG-Cb-4063 is characterized by a triangular to rounded section and an elongated depression passing through its length. The drill core (Fig. S1) was extracted from an area which appeared to be preserving the outermost cortical surface since deposition time, as suggested by external texture and by possible scavenging marks (2021, *pers. observation*) on it.

**BRSMG-Cg-2488 R 101 from Lilstock**

BRSMG-Cg-2488 represents the almost complete putative large ichthyosaurian surangular described by Lomax *et al.* (2018) (Fig. S2C). It comes from the top of the Westbury Formation of Lilstock. The bone is divided into five connecting segments (Lomax *et al.* 2018) (Fig. S2C). Although showing areas of superficial erosion (Fig. S2E), the outermost layers are still unworn in some regions. Following the homology hypothesis between the 5^th^ rostral fragment (BRSMG-Cg-2488 R 101) and BRSMG-Cb-3869, proposed by Lomax *et al.* (2018), a drill-core was extracted trying to match the position of the drill core previously taken from BRSMG-Cb-3869 relative to the presence of an elongated furrow (Fig. S1).

**KULeuven PLV-1964 from Autun: aff. Shastasauridae**

The specimen consists of two large fragments (a massive j-shaped one and a smaller cylindrical one) coming from the Rhaetian sediments of the Autun area, central France (Fischer *et al.* 2014) (Fig. S2). The two pieces measure respectively 103 mm and 67 mm in maximum width and 400 and 290 mm in length. The two pieces were previously subjected to anatomical description and attributed to a single individual. Fischer *et al.* (2014) identified these fossils as a piece of splenial (the larger fossil) and a portion of the dentary (the smaller fossil), while Lomax *et al.* (2018) proposed the latter to be a piece of surangular. Despite the different anatomical placement, both studies agree on a Shastasauridae affinity of PLV-1964 (Fischer *et al.* 2014; Lomax *et al.* 2018). The smaller cylindrical piece was subjected to core drilling, resulting in cross and longitudinal sections (Table 1, Fig. S1), while a small cortical fragment detached from the specimen and was sectioned transversely to the bone long axis. View (Table 1).

# **Bonenburg material: Tetrapoda indet.**

The material coming from the Rhaetian Exter Formation of Bonenburg is composed of several fragmentary pieces collected since 2015 by the joint efforts of WMNM and IGPB. The fragments were all found in bonebeds 2 and 3 exposed in the Bonenburg clay pit (Sander *et al.* 2016). These bonebeds are situated in the dark shales of the Contorta Beds in the middle of the Exter Formation and recently were dated palynologically as late middle Rhaetian (Bonebed 2) and early late Rhaetian (Bonenbed 3), respectively (Gravendyck *et al.* 2020). Most of these fragments are relatively small (one to three cm in diameter, Fig S4A), and block-like in shape, and clearly represent the cortical fragment of much larger bones (long bones, dermatocranial elements, ribs). Due to the fragmentary nature and the lack of clear diagnostic characters, these fossils were stored in IGPB collection as cortex fragments, awaiting further study (Table 1). Because of its abundance, this material offers the possibility to perform complete destructive sampling through full cross and longitudinal sections (Table 1). Therefore, complete sections, both transverse and longitudinal were performed on this material.

**WMNM P88133**

This specimen is the largest cortical bone fragment from the Bonenburg collection (Table 1; Fig. S3A); morphologically, the specimen represents either a large skull bone or the fragment of a large bone shaft. A complete cross section and partial longitudinal section were produced from this specimen (Fig. S3B).

**WMNM P-uncatalogued**

This specimen is similar to WMNM P88133, but less complete. It shows a rounded natural surface on the external and upper(?) margin (Fig. S5A), a clear broken surface on the lower margin and an abraded surface on the inner margin, indicating the absence of bone material, like in WMNM P88133. Morphologically, the specimen could represent a fragment of a large skull bone or rib.

**WMNM P88130-P88132, WMNM P88134-88144**

A collection of various small fragments of different size and shapes was studied as representative of the bulk of similar fragmentary remains often found in Bonenburg (Table 1). Cross and longitudinal sections were performed to obtain a broad histological overview of the collection (Fig. S4).

# ***Shastasaurus sikanniensis* RTMP-1994-378-0002 from the middle Norian of British Columbia**

The sampled material belonging to *Shastasaurus sikanniensis* (Table 1, Figs 5, S2, S4F, G), from the middle Norian of British Columbia (Canada) amounts to four thin sections (two for each sample taken). The current recognized status of *S.sikanniensis* as the largest articulated ichthyosaurs yet described (Nicholls and Manabe 2004) led the choice of the use of this taxon as control despite the important difference in time (~10-15 my) that separates *S.sikanniensis* from the rest of the specimens, which are Rhaetian in age.

RTMP-1994-378-0002 (*S. sikanniensis* holotype) was sampled by staff of the Royal Tyrrell Museum, where it is currently on exhibit. Sample locations on the surangular and splenial are indicated in Fig. S2A, B. Sections from the splenial (Fig. S2F) are shown in Figs 5A and S6G and those of surangular in Fig. S6F.

RTMP-1994-378-0002 is a large two-thirds completed articulated skeleton, the holotype  of *S. sikanniensis*, estimated to be 21 m in length based on observations during excavation (Nicholls & Manabe 2004)*.* The skeleton is characterized by spatially variable preservation, even in the same bone, from heavily flattened and crushed to more intact elements, regardless of microanatomy and histology. The skull in particular, although articulated, shows heavy compression and surface damages in some places (Nicholls & Manabe 2004), expected to influence the preservation of the microstructure observable in the thin sections.

# Appendix S1. References

Lomax, D. R., P. De la Salle, J. A. Massare, and R. Gallois. 2018. A giant Late Triassic ichthyosaur from the UK and a reinterpretation of the Aust Cliff ‘dinosaurian’ bones. *PLoS ONE*, 13, 1– 16.

Galton, P. M. 2005. Bones of large dinosaurs (Prosauropoda and Stegosauria) from the Rhaetic Bone Bed (Upper Triassic) of Aust Cliff, southwest England. *Revue de Paleobiologie*, 24, 51– 74.

Gravendyck, J., M. Schobben, J. B. Bachelier, and W. M. Kürschner. 2020. Macroecological patterns of the terrestrial vegetation history during the end-Triassic biotic crisis in the central European Basin: A palynological study of the Bonenburg section (NW-Germany) and its supra-regional implications. *Global and Planetary Change*, 194, 103286.

Fischer, V., H. Cappetta, P. Vincent, G. Garcia, S. Goolaerts, J. E. Martin, D. Roggero, and Valentin, X. 2014. Ichthyosaurs from the French Rhaetian indicate a severe turnover across the Triassic–Jurassic boundary. *Naturwissenschaften*, 101, 1027– 1040.

Redelstorff, R., P. M. Sander, and P. M. Galton. 2014. Unique bone histology in partial large bone shafts from Upper Triassic of Aust Cliff, England: An early independent experiment in gigantism. Acta Palaeontologica Polonica 59, 607– 615.

Nicholls, E. L., and M. Manabe. 2004. Giant ichthyosaurs of the Triassic-A new species of *Shonisaurus* from the Pardonet Formation (Norian, Late Triassic) of British Columbia. *Journal of Vertebrate Paleontology* 24, 838– 849.

Sander, P. M., T. Wintrich, A. H. Schwermann, and R. Kindlimann. 2016. Die paläontologische Grabung in der Rhät-Lias-Tongrube der Fa. Lücking bei Warburg-Bonenburg (Kr. Höxter) im Frühjahr 2015. *Geologie und Paläontologie in Westfalen* 88, 11– 37.
