# Supplementary material for: The dinosaurs that weren’t: osteohistology supports giant ichthyosaur affinity of enigmatic large bone segments from the European Rhaetian
Source: PeerJ. 2024 Apr 9;12:e17060. doi: 10.7717/peerj.17060 (PMC11011611; doi:10.7717/peerj.17060)
Supplement: Supplemental Information 10 [file peerj-12-17060-s010.docx]

**Supplementary article 2**

**On the occurrence of similar coarse fibered tissues to IFM**

A tissue possibly similar to PIFT is the unusual parallel-fibered bone (UPFT, see Fig. S7C) described by Houssaye et al. (2013) in another marine reptile clade, mosasaurs. However, neither the description nor the illustrations of UPFT by Houssaye et al. (2013) are sufficient to understand UPFT. From what can be gleaned, UPFT seems to lack the abundant, distinctive and highly organized intrinsic fibers observed by us in IFM (see Fig. S7F). More recently, Klein & Griebeler (2016) and Klein et al. (2023) described coarse parallel-fibered bone (CPFB) in Triassic marine reptiles, the basal thalattosaur *Askeptosaurus* (Fig. S7D, E) and the nothosauroid *Simosaurus* (Fig. S7H, I). The matrix of CPFB probably is the closest we have observed to IFM.

The bone tissue of *Askeptosaurus* has abundant intrinsic fibers in its long bone primary periosteal tissue (hence coarse parallel-fibered bone, CPFB). The vertebrae, ribs and gastralia of the same individuals also have a very fibrous matrix, but there is an obvious, if difficult to quantify, contribution by extrinsic fibers, i.e., Sharpey's fibers. Finally, CPFB is a type of parallel-fibered bone tissue. Even in the femora, as the largest bones, the tissue either entirely lacks primary osteons (in the smaller but older of the two sampled *Askeptosaurus* individuals, PIMUZ T4839) or only locally has radial primary osteons (in the larger but younger of the two sampled *Askeptosaurus* individuals, PIMUZ T4840). The intrinsic fibers in *Askeptosaurus* CPFB (Fig. S7D) also lack the great regularity provided by the uniform honeycomb pattern of the material we studied. The honeycomb pattern is only locally developed in *Askeptosaurus*. Thus *Askeptosaurus* primary periosteal cortical bone lacks the defining features of PIFT, i.e., the abundant longitudinal primary osteons set in a woven matrix of highly organized longitudinal intrinsic fibers (i.e., IFM). It is interesting that a tissue that clearly has an important intrinsic fiber contribution should be found in a thalattosaur, considering certain histological similarities between ichthyosaurs and thalattosaurs (Klein et al. 2023) and the possibility that the two clades are sister taxa (Chen et al. 2014).

The case is somewhat different in *Simosaurus* as described by Klein & Griebeler (2016). The CPFB is (1) not in the periosteal territory (unlike PIFT, as the acronym already indicates) but in the endosteal territory, and (2) CPFB is an exception in *Simosaurus*, not the rule. Only one out of five putative *Simosaurus* specimens, SMNS 91983, showed this tissue. The authors were puzzled by this single specimen and also note how much it differs from the other four in other aspects of its histology (Klein & Griebeler 2016). We suspect that the fragmentary femur in question may actually pertain to another taxon than *Simosaurus*. This is also suggested by the structure of the outer bone surface that is rather different (Klein & Griebeler 2016, fig. 2c) compared to the other *Simosaurus* specimens (Klein & Griebeler 2016, fig. 2a,b,d,e). The bone tissue in SMNS 91983 is obviously much more vascularized, and the arrangement of the vascular canals is rather similar to that of plesiosaurs. There, the canals are radial at midshaft and then fan out towards the epiphyses (Liebe & Hurum 2012; Sander & Wintrich 2021).

Coarse crossed mineralized fibers similar to those observed in our samples are often referred to as interwoven structural fibers (ISF) and are characteristic of ossified tendons (Fig. S7G, J) and osteoderms, usually associated with metaplastic ossification (Organ & Adams 2005; Klein, Christian & Sander 2012; Vickaryous, Meldrum & Russell 2015; Scheyer, Syromyatnikova & Danilov 2017; Buffrénil & Quilhac 2021a, fig. 8.7h; Surmik *et al.* 2023). It is important to note that the developmental origin of metaplastic bones is yet to be fully understood and that “metaplasia” can encompass a variety of different cellular mechanisms (Horner, Woodward & Bailleul 2016; Buffrénil & Zylberberg 2021). Furthermore metaplastic bone can occur in combination with other, more common bone types such as periosteal bone (Organ & Adams 2005; Klein, Christian & Sander. 2012; Surmik *et al.* 2023).

Several histological studies of non-amniote tetrapods show the coarse fibers bundles as well, both in cranial bone cortices of the temnospondyl *Metoposaurus* (Gruntmejer, Konietzko-Meier & Bodzioch 2016; Gruntmejer, Bodzioch & Konietzko-Meier*.* 2021) (Fig. S7K) and in the humerus periosteal cortex of an indeterminate cyclotosaurian temnospondyl (Konietzko-Meier *et al.* 2018 fig. 3g) (Fig. S7L). While the latter study does not address the developmental origin of such structures (only mentioning them as coarse fibers), the former describes them as ISF and present them as proof of metaplastic developmental process being involved in the formation of dermatocranial bones of dermal origin.

In the other cases, the resemblance is clearly superficial because the fibrous tissue in question originated from the incorporation of extrinsic (i.e., Sharpey’s) fibers into the primary periosteal tissue. This is particularly true for any rib sample where the intercostal musculature leaves ample traces of its insertion.

References

Buffrénil V. de and Quihlac A. 2021*a*. Bone Tissue Types: A Brief Account of Currently Used Categories. 183–188. *In* de Buffrénil V., de Ricqlès A.J., Zylberberg L. and Padian K. (Eds.). (2021). *Vertebrate Skeletal Histology and Paleohistology* (1st ed.). Boca Raton: CRC Press. 838 pp. https://doi.org/10.1201/9781351189590

Chen X-h., Motani R., Cheng L., Jiang D-y. and Rieppel O. 2014. The enigmatic marine reptile *Nanchangosaurus* from the Lower Triassic of Hubei, China and the phylogenetic affinities of Hupehsuchia. *PLoS ONE* 9:e102361. 10.1371/journal.pone.0102361

Gruntmeijer K., Konietzko-Meier D., Bodzioch A. 2016. Cranial bone histology of *Metoposaurus krasiejowensis* (Amphibia, Temnospondyli) from the Late Triassic of Poland. *PeerJ*, 4,e2685; DOI 10.7717/peerj.2685.

Gruntmeijer K., Bodzioch A., Konietzko-Meier D. 2021. Mandible histology in *Metoposaurus krasiejowensis* (Temnospondyli, Stereospondyli) from the Upper Triassic of Poland. *PeerJ*, 9,e12218; DOI 10.7717/peerj.12218.

Houssaye A., Lindgren J., Pellegrini R., Lee A.H., Germain D. and Polcyn M.J., 2013. Microanatomical and Histological Features in the Long Bones of Mosasaurine Mosasaurs (Reptilia, Squamata) – Implications for Aquatic Adaptation and Growth Rates. *PLoS ONE* 8(10): e76741. doi:10.1371/journal.pone.0076741

Klein N. and Griebeler E.M. 2016. Bone histology, microanatomy, and growth of the nothosauroid *Simosaurus gaillardoti* (Sauropterygia) from the Upper Muschelkalk of southern Germany/Baden-Württemberg. *Comptes Rendus Palevol*, 15, 142– 162. https://doi.org/10.1016/j.crpv.2015.02.009

Klein N., Christian A. and Sander P. M. 2012. Histology shows that elongated neck ribs in sauropod dinosaurs are ossified tendons. *Biology Letters*, 8 1032– 1035.

Klein N., Sander P. M., Liu J., Druckenmiller P.,Metz E. T., Kelley N. P. and Scheyer T. M. 2023. Comparative bone histology of two thalattosaurians (Diapsida: Thalattosauria): *Askeptosaurus* italicus from the Alpine Triassic (Middle Triassic) and a Thalattosauroidea indet. from the Carnian of Oregon (Late Triassic). *Swiss Journal of Palaeontology*, 142, 15 https://doi.org/10.1186/s13358-023-00277-3.

Konietzko-Meier D. and Sander P. M. 2013. Histology of long bones of *Metoposaurus diagnosticus krasiejowensis* (Temnospondyli) from the Late Triassic of Krasiejów (Opole, Silesia Region). *Journal of Vertebrate Paleontology*, 33, 1003– 1018.

Konietzko-Meier D., Werner J. D., Wintrich T. and Sander P. M. 2018. A large temnospondyl humerus from the Rhaetian (Late Triassic) of Bonenburg (Westphalia, Germany) and its implications for temnospondyl extinction. *Journal of Iberian Geology*, 45, 287– 300.

Liebe L. and Hurum J.H. 2012. Gross internal structure and microstructure of plesiosaur limb bones from the Late Jurassic, central Spitsbergen. *Norwegian Journal of Geology*, 92, 285-309. ISSN 029-196X.

Organ C. and Adams J. 2005. The histology of ossified tendons in dinosaurs. *Journal of Vertebrate Paleontology*, 25(3), 602– 613.

Sander P. M. and Wintrich T. 2021. Sauropterygia: Histology of Plesiosauria. 444– 455. *In* de Buffrénil V., de Ricqlès A.J., Zylberberg L., & Padian K. (Eds.). (2021). *Vertebrate Skeletal Histology and Paleohistology* (1st ed.). Boca Raton: CRC Press. 838 pp.

Scheyer T. M., Syromyatnikova E. V. and Danilov I. G. 2017. Turtle shell bone and osteoderm histology of Mesozoic and Cenozoic stem-trionychian Adocidae and Nanhsiungchelyidae (Cryptodira: Adocusia) from Central Asia, Mongolia, and North America. *Fossil Record*, 20, 69– 85.

Surmik D., Słowiak-Morkovina J., Szczygielski T., Wojtyniak M., Środek D., Dulski M., Balin K., Krzykawski T. and Pawlicki R. 2023. The first record of fossilized soft parts in ossified tendons and implications for the understanding of tendon mineralization. *Zoological Journal of the Linnean Society*, XX, 1– 20.

Vickaryous M. K., Meldrum G. and Russell A. P. 2015. Armored geckos: A histological investigation of osteoderm development in *Tarentola* (Phyllodactylidae) and *Gekko* (Gekkonidae) with comments on their regeneration and inferred function. *Journal of Morphology*, 276, 1345– 1357.
